# Recruitment and rejoining of remote double-strand DNA breaks for enhanced and precise chromosome editing

**DOI:** 10.1186/s13059-025-03523-8

**Published:** 2025-03-11

**Authors:** Mingyao Wang, Pengchong Fu, Ziheng Chen, Xiangnan Wang, Hanhui Ma, Xuedi Zhang, Guanjun Gao

**Affiliations:** 1 Gene Editing Center, School of Life Science and Technology, ShanghaiTech University, 393 Middle Huaxia Road, Pudong, Shanghai, 201210 China; 2 Department of Cell Biology, School of Basic Medical Sciences, Suzhou Medical College, Soochow University, Suzhou, Jiangsu Province 215123 China

**Keywords:** CRISPR-Cas9, Chromosome rearrangement, Homologous recombination, Double-strand DNA break repair

## Abstract

Chromosomal rearrangements, such as translocations, deletions, and inversions, underlie numerous genetic diseases and cancers, yet precise engineering of these rearrangements remains challenging. Here, we present a CRISPR-based homologous recombination-mediated rearrangement (HRMR) strategy that leverages homologous donor templates to align and repair broken chromosome ends. HRMR improves efficiency by approximately 80-fold compared to non-homologous end joining, achieving over 95% homologous recombination. Validated across multiple loci and cell lines, HRMR enables efficient and accurate chromosomal rearrangements. Live-cell imaging reveals that homologous donors mediate chromosome end proximity, enhancing rearrangement efficiency. Thus, HRMR provides a powerful tool for disease modeling, chromosomal biology, and therapeutic applications.

**Supplementary Information:**

The online version contains supplementary material available at 10.1186/s13059-025-03523-8.

## Background

Chromosome rearrangements including translocations, inversions, and large deletions are frequent drivers of cancers and progression. These aberrations can dysregulate oncogenes or deactivate tumor suppressors by rewiring chromatin structure and gene expression [1–4]. As more disease-associated lesions are uncovered through sequencing efforts, modeling these events in human cells is essential to elucidate mechanisms linking chromosomal instability to cancer evolution [5–7]. However, efficiently engineering precise rearrangement has remained challenging.

The CRISPR-Cas9 system enables introducing targeted DNA double-strand breaks (DSBs) but jointing distal DSBs on separate chromosomes via non-homologous end joining (NHEJ) is extremely inefficient due to the rarity of DSB end interactions [6–14]. Alternative approaches like prime editing can generate rearrangements with improved accuracy but lower efficiency compared to NHEJ pathway [7, 15, 16]. Hence, the development of efficient and precise chromosome editing tools remains an imperative objective. Conventional genome-editing techniques such as exogenous knock-in frequently rely on homologous recombination (HR) pathway, but the challenge of finding matching sequences within the vast genome has been a barrier [17–19]. Notably, mechanisms related to homologous recombination, such as multi-invasion-induced rearrangement (MIR) in yeast, have been described in the field of DNA repair, where complex rearrangements occur through simultaneous invasions of broken DNA ends into intact donor molecules [19]. Building on these insights, we hypothesized that providing additional homologous donors to recruit and align broken chromosome ends could promote repair through homologous recombination, enhancing rearrangement frequencies.

Here, we developed a homologous recombination-mediated rearrangement (HRMR) strategy to model pathological lesions and novel events. We show HRMR stimulates recombination between distal DNA cuts by orders of magnitude versus non-homologous end joining alone. We induce translocations, inversions, and large deletions across over 10 loci in the human genome. We also characterize critical parameters for optimal donor design. This chromosome engineering approach will facilitate disease modeling, provide insights into genome evolution, and enable therapeutic editing of deleterious rearrangements.

## Results

### Homologous recombination-mediated rearrangement (HRMR) strategy

In recent years, genome editing has seen significant progress, with technologies like base editing, prime editing, and the use of Cas9/gRNA allowing precise genetic modifications, encompassing base substitution, small fragment insertion, deletion, and replacement. While Cas9 targeting multiple DNA sites with separate sgRNAs efficiently generates DNA fragment deletion under 10 kb in size [7, 16, 17], larger fragment edits, such as megabase (MB) scale chromosome translocations or fragment deletions, encounter decreased efficiency. This reduced efficiency can be primarily ascribed to the physical separation between distant chromosome sites, which hinders the binding and interaction of DNA repair proteins, such as KU [20].

**Fig. 1:**
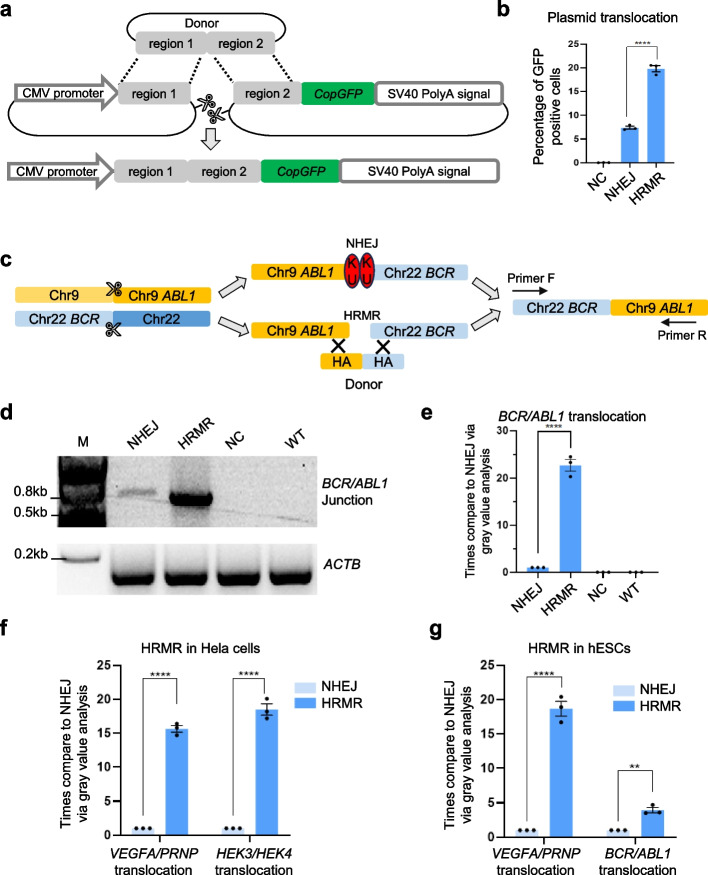
Overview of homologous recombination-mediated rearrangement (HRMR) and feasibility in different genome sites and cell types. **a** Schematic diagram of plasmid translocation within two plasmids containing CMV promoter and CopGFP-SV40polyA signal, respectively. Two sgRNAs targeted region 1 and region 2 induce plasmid double-strand breaks. Homologous donor was introduced to promote plasmid translocation. **b** Quantifications of the percentage of GFP positive cells by FACS. NC represents the HRMR control without sgRNA. Independent biological replicates were performed (*n* = 3) and error bars show the s.e.m. **c** Schematic diagram of homologous recombination-mediated chromosome rearrangement at *BCR/ABL1* loci. Two sgRNAs targeted *BCR* on chr22 and *ABL1* on chr9 were used to introduce double-strand DNA break. Specified primers were used to detect rearranged chromosomes. **d** The agarose gel image of the translocation between *BCR* on chr22 and *ABL1* on chr9 was detected by PCR analysis with primer flanking each side of translocation. The *ACTB* gene was amplified to ensure consistent genomic usage. **e** Quantifications of bands gray density to determine the relative translocation efficiency at *BCR/ABL1* loci in HEK293T cells. NC represents the HRMR control without sgRNA. Independent biological replicates were performed (*n* = 3) and error bars show the s.e.m. The feasibility of HRMR in Hela cells (**f**) and hESCs (**g**) at endogenous genome loci including random rearrangement and pathological rearrangement. Relative translocation efficiency was determined via quantification of bands gray density. Independent biological replicates were performed (*n* = 3) and error bars show the s.e.m

To determine if homologous donors could stimulate recombination between distal DNA cuts, we developed a split GFP reporter plasmid that undergoes recombination upon introducing two distal DSBs, restoring GFP expression. Three days after co-transfecting of Cas9, sgRNAs targeting the reporter gene, and a donor plasmid into wild-type HEK293T cells, flow cytometry analysis revealed that the proportion of GFP-positive cells reached 21%, representing a threefold increase compared to the donor-free control group (Fig. 1a, b). This provides initial evidence that supplying homologous donors can promote recombination between separate DNA sequences. The donor likely provides homology to help recruit and align the broken plasmid ends, facilitating their rejoining through homologous recombination repair. The sequencing results demonstrate the proportions of homologous recombination- and NHEJ-derived GFP-positive cells. When providing a homology arm plasmid, about 77% of GFP-positive cells exhibited intact “region 1-region 2” sequences (precise translocation) consistent with donor, while about 23% contained indels or other disruptions characteristic of NHEJ (Additional file 1: Fig. S1a, b). These findings confirm that both repair pathways contribute to GFP-positive cell generation, with homologous recombination accounting for majority of the events.

Next, we attempted to investigate whether the presence of homologous arms has a positive effect on dsDNA recombination. We developed a split GFP dsDNA reporter system (Additional file 1: Fig. S1c) and results showed that introduction of homologous arms can improve the efficiency of dsDNA recombination, and sanger sequencing shows that HR is the main repair mode of dsDNA recombination (Additional file 1: Fig. S1d, e, f).

### Homologous donors enable efficient chromosome rearrangement

We next tested if HRMR could stimulate rearrangements at endogenous chromosomal loci. We first targeted the *BCR* and *ABL1* loci to model the Philadelphia chromosome translocation associated with chronic myeloid leukemia [1, 10, 21] (Fig. 1c). Utilizing two sgRNA targeting *ABL1* and *BCR* along with Cas9, we introduce two double-strand DNA breaks, systematically comparing the translocation efficiency between *ABL1* and *BCR* with and without the incorporation of the homologous donor. PCR amplification across the derivative chromosome junction in HRMR revealed a about 20-fold increase in recombination efficiency with the homologous donor compared to NHEJ alone (Fig. 1d, e).

To further affirm the versatility of HRMR across various genomic sites, we tested this approach on 11 different chromosome rearrangements, encompassing both random and pathological chromosome translocation, transversion, and large fragment knockouts. The results unequivocally demonstrated that the presence of a homologous donor significantly enhances chromosome rearrangement efficiency, resulting in an increase of up to 22-fold (Additional file 1: Figs. S2, S3). Next, we assessed HRMR at endogenous sites in two additional human cell lines. In Hela cells, HRMR achieved a remarkable 15- to 20-fold increase in efficiency when compared to NHEJ, particularly in generating two random chromosome translocations (Fig. 1f). Similarly, in human embryonic stem cells (hESCs), HRMR displayed significant improvements, achieving an 18-fold increase in efficiency for random chromosome translocation and a fourfold increase for pathological chromosome translocation when compared to NHEJ (Fig. 1g). Together, these results establish that HRMR allows efficient engineering of chromosomal aberrations found in cancers and de novo rearrangements, with broad applicability across multiple human cell types.

### Enhancing HRMR efficiency through optimization

To optimize the efficiency of HRMR approach, we embarked on a systematic evaluation of the lengths of homologous arms. Specifically, we assessed arm lengths ranging from 30 to 500 base pairs (bp) at the *BCR/ABL1* translocation sites (Additional file 1: Fig. S4a, b) and insertion lengths varying from 18 to 400 bp at *VEGFA/PRNP* translocation sites (Additional file 1: Fig. S4c, d, e). Notably, as the length of the homologous arm decreased, the efficiency of chromosome rearrangement diminished. However, we observed no significant decrease in chromosome rearrangement efficiency when employing homologous arms of 200 bp and 500 bp. In contrast, homologous donor with HAs below 120 bp exhibited significantly lower editing efficiency. These results suggested that homologous arm lengths of 200 bp are sufficient for efficient chromosome rearrangement.

To explore whether multiple types of homologous donors might interfere with each other, we conducted experiments where two sgRNAs were used to simultaneously target *ABL1* and *BCR*, leading to double-stranded breaks on chr9 and chr22. This kind of breaks could initiate two forms of translocation, namely, *BCR/ABL1* and *ABL1*/BCR translocations (Additional file 1: Fig. S5a). We attempt to simultaneously promote the *BCR/ABL1* translocation and *ABL1/BCR* translocation using two homologous donors. We found that when the homologous donor aimed at enhancing *BCR/ABL1* translocation was utilized, the efficiency of *BCR/ABL1* translocation was substantially elevated, while *ABL1/BCR* translocation efficiency displayed a modest improvement (Additional file 1: Fig. S5b, c). This pattern held true when we used a different homologous donor. However, when two homologous donors were employed simultaneously, both *BCR/ABL1* and *ABL1/BCR* translocation efficiencies were significantly enhanced (Additional file 1: Fig. S5b, c).

**Fig. 2:**
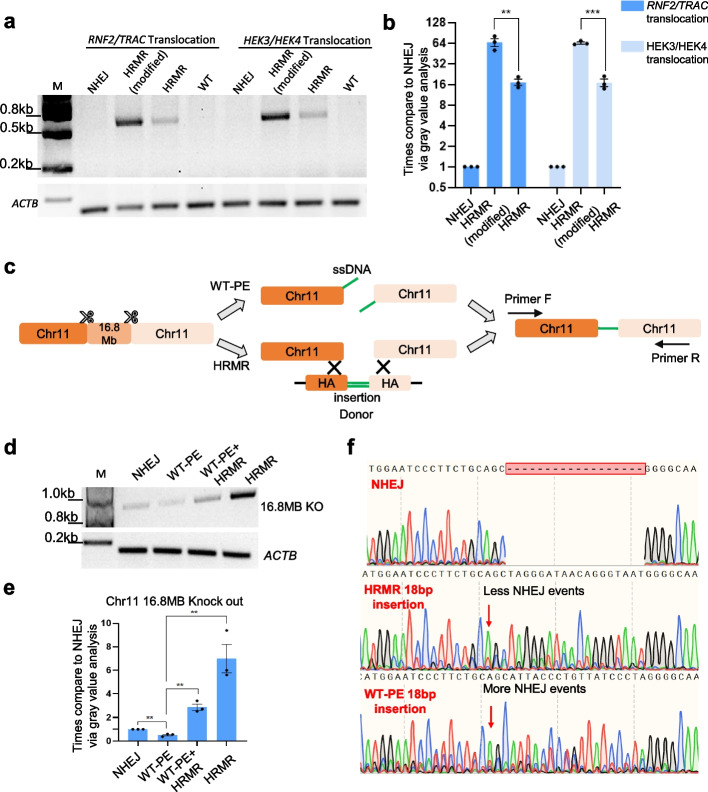
Optimizing HRMR and comparing it with WT-PE-mediated chromosome rearrangement. **a** The agarose gel image of *RNF2/TRAC* and *HEK3/HEK4* site translocation in HEK293T cells. The junction was detected by PCR analysis with primer flanking each side of translocation. The *ACTB* gene was amplified to ensure consistent genomic usage. **b** Relative translocation efficiency at *RNF2/TRAC* and *HEK3/HEK4* site using HRMR or HRMR (modified) in HEK293T cells via quantification of bands gray density. Independent biological replicates were performed (*n* = 3) and error bars show the s.e.m. **c** Schematic diagram of WT-PE mediated chromosome rearrangement and homologous recombination-mediated chromosome rearrangement. **d** The agarose gel image of the 16.8 MB knock out on chr11 was detected by PCR analysis with primer flanking each side of rearrangement. The *ACTB* gene was amplified to ensure consistent genomic usage. **e** Quantifications of bands gray density and determination the relative knock-out efficiency. Independent biological replicates were performed (*n* = 3) and error bars show the s.e.m. **f** Three sanger sequencing chromatograms showed different repair outcomes with red arrow points to the nested peaks

To further optimize HRMR efficiency, we hypothesized that increasing the nuclear concentration of homologous donors would enhance their interaction probability with matching chromosomal sequences. We introduced a nuclear import sequence (NIS) to the donors to promote nuclear uptake [22]. We found that donors containing the NIS showed 3–fourfold higher chromosome translocation efficiency compared to donors without NIS. Relative to NHEJ without any homologous donor, the NIS-containing donors improved efficiency up to 60-fold (Fig. 2a, b). These results highlight that concentrate donors in the nucleus may enable efficient chromosome arrangement. This modified HRMR strategy will be used for all subsequent experiments.

### Efficient large fragment deletion by HRMR compared to WT-PE

We compared the efficiency of homologous recombination-mediated repair (HRMR) with wild-type prime editing (WT-PE) for generating large genomic deletions. WT-PE is a CRISPR-based method that uses a wild-type Cas9 fused to a reverse transcriptase (RT) and a prime editing guide RNA (pegRNA) to directly introduce precise sequence changes without requiring a DNA template. Paired pegRNAs direct Cas9-RT to create two DNA nicks, while the reverse transcriptase incorporates new sequences encoded by the pegRNAs, enabling precise deletions or modifications without relying on homology-directed repair (HDR) [7, 16] (Fig. 2c). Modeling a 16.8 Mb deletion in HEK293T cells revealed HRMR achieved sixfold higher efficiency than non-homologous end joining, while WT-PE showed 0.5-fold lower efficiency than NHEJ (Fig. 2d, e). Sanger sequencing confirmed both strategies could incorporate exogenous insertions at the deletion junction, unlike NHEJ. However, the relatively low baseline noise in the chromatogram for pooled deletion events (Fig. 2f) reflects the minimal indel formation in specific chromosomal rearrangements, such as the 16.8 Mb deletion on chr11. This is because NHEJ-mediated jointing of fragments does not necessarily result in substantial indels at the junctions. Additionally, the observed indels in HRMR and WT-PE products likely arise from a mixture of HDR and NHEJ repair events, highlighting the repair pathway heterogeneity underlying these rearrangements. Notably, HRMR resulted in fewer indels, indicating it is a more effective and accurate repair mechanism (Fig. 2f). Together, these results demonstrate HRMR mediates large chromosomal deletions more efficiently than current prime editing approaches while also maintaining higher fidelity. The ability to generate precise megabase-scale deletions further expands the versatility of HRMR for diverse chromosome engineering applications.

### HRMR is a precise pathway for engineering chromosomal rearrangements

**Fig. 3:**
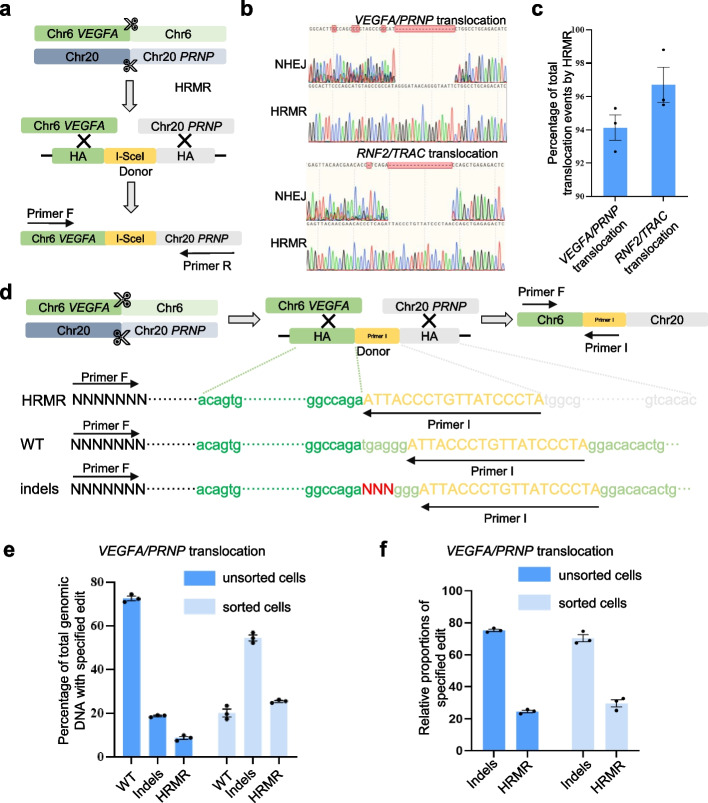
Quantifying the accuracy and efficiency of HRMR. **a** Schematic diagram of I-SceI recognition sequence insertion to quantifying the accuracy of HRMR. **b** Sanger sequencing chromatograms showed different repair outcomes with varying degrees of nested peaks. Upper panel, the Sanger sequencing chromatograms of *VEGFA/PRNP* translocation sites. Lower panel, the Sanger sequencing chromatograms of *RNF2/TRAC* translocation sites. **c** HTSs of *VEGFA/PRNP* translocation and *RNF2/TRAC* translocation showed percentage of total translocation events by HRMR. Independent biological replicates were performed (*n* = 3) and error bars show the s.e.m. **d** Schematic diagram of primer insertion to quantifying the accuracy of homologous recombination-mediated chromosome rearrangement. Primer I sequence was same with sequence in *VEGFA* site. Primer F and primer I were used to amplify *VEGFA* genomic sites (WT) and other two edit outcomes were shown, including HRMR and indels. **e** HTSs of the *VEGFA* genomic site, amplified using primer F and primer I, revealed the percentage of total genomic DNA containing the specified edits in unsorted and sorted cells. **f** In the sequencing data shown in (**e**), after excluding wild-type (WT) reads, the relative proportions of indels and HRMR were calculated

We next sought to further evaluate the accuracy of HRMR compared to NHEJ. We introduced an I-SceI restriction site into the homologous donors for two rearrangement models—*VEGFA/PRNP* translocation and *RNF2/TRAC* translocation (Fig. 3a, Fig. S6a). PCR amplification of genomic DNA flanking the targeted loci, followed by I-SceI digestion, revealed a large proportion of HRMR-mediated rearrangements contained the I-SceI site, indicating high fidelity (Additional file 1: Fig. S6b, c). Sanger sequencing confirmed the majority of HRMR events occurred precisely through the homologous donors, while NHEJ caused indels at the junctions (Fig. 3b). The overwhelming presence of precise HRMR junctions effectively masks the lower-frequency NHEJ events, contributing to the minimal baseline noise observed in the Sanger sequencing chromatograms for pooled translocation events (Fig. 3a, b). High-throughput sequencing (HTS) quantified HRMR-mediated precise translocations were up to 50-fold higher than NHEJ (the percentage of translocation events repaired by HRMR > 95%), establishing HRMR as the predominant pathway for accurate rejoining (Fig. 3c). Together, these results demonstrate HRMR enables high-precision chromosome engineering through the homology-directed repair pathway. Incorporating unique sequences in donors provides a robust molecular strategy to enrich for desired events over background mutagenesis, indicating the ability to perform complex rearrangements with such accuracy is a key advantage of HRMR over existing methods.

### Primer-insertion assay quantifies HRMR efficiency

Although the addition of homologous donors enhanced chromosome rearrangement efficiency, the precise recombination frequency remained unknown. To address this, we developed a primer-insertion strategy to quantify the percentage of HRMR-mediated translocations at the *VEGFA/PRNP* locus (Fig. 3d, Additional file 1: Fig. S7). This strategy utilizes primer I, inserted into the homologous donor sequence, which binds near the sgRNA cleavage site at the *VEGFA* locus. Primer I serves as a unique marker for HRMR events, allowing simultaneous detection of unedited chromosomes, indels, and HRMR-mediated translocations at the *VEGFA* locus (Fig. 3d, Additional file 1: Fig. S7). To distinguish between repair outcomes, we performed genomic amplification using primer F (upstream of the *VEGFA* DSB) and primer I. This allowed us to identify and quantify wild-type (WT) sequences, NHEJ-mediated indels, HRMR-mediated translocations, and recombination events mediated by HR (with 23 bp primer I serving as the right homology arm). High-throughput sequencing was used for precise quantification of these events. In unsorted transfected cells, sequencing revealed ~ 70% wild-type alleles, ~ 20% NHEJ events, and ~ 10% HRMR-mediated translocations (Fig. 3e, Additional file 1: Fig. S8). Rare recombination events involving 23 bp primer I homology arm were detected but were negligible due to their minimal contribution to the overall repair outcomes. In sorted transfected cells, where DSB repair occurred more efficiently, sequencing results showed ~ 20% wild-type alleles, ~ 59% indels, and ~ 21% HRMR-mediated translocations (Fig. 3e).

Since WT sequences primarily reflect genomic DNA that was not cleaved, we focused exclusively on cleavage events. Excluding WT, approximately 75% of the amplified products corresponded to indels formed by the direct rejoining of the *VEGFA* gene following cleavage, while the remaining 25% represented HRMR-mediated chromosomal translocations (Fig. 3f). These results indicate, although chromosomal self-ligation (indels) at the DNA break sites constitutes the majority, HRMR occurs in a substantial fraction of chromosomes when HR donors are provided.

Further analysis of the translocation-specific events, conducted through high-throughput sequencing of the genome amplified using primer F and primer R (Additional file 1: Fig. S7), revealed that only 5% of translocations were mediated by NHEJ, whereas 95% were mediated by homologous recombination (Fig. 3c) These findings demonstrate that HR-mediated translocations make up the majority of all translocation events when homologous donors are provided. Overall, when homologous donors are provided, chromosomal translocations are primarily mediated by homologous recombination, accounting for approximately 21% of all successfully transfected cells at VEGFA/PRNP locus (Fig. 3f).

### Misaligned sgRNAs or donors retain partial HRMR activity

**Fig. 4:**
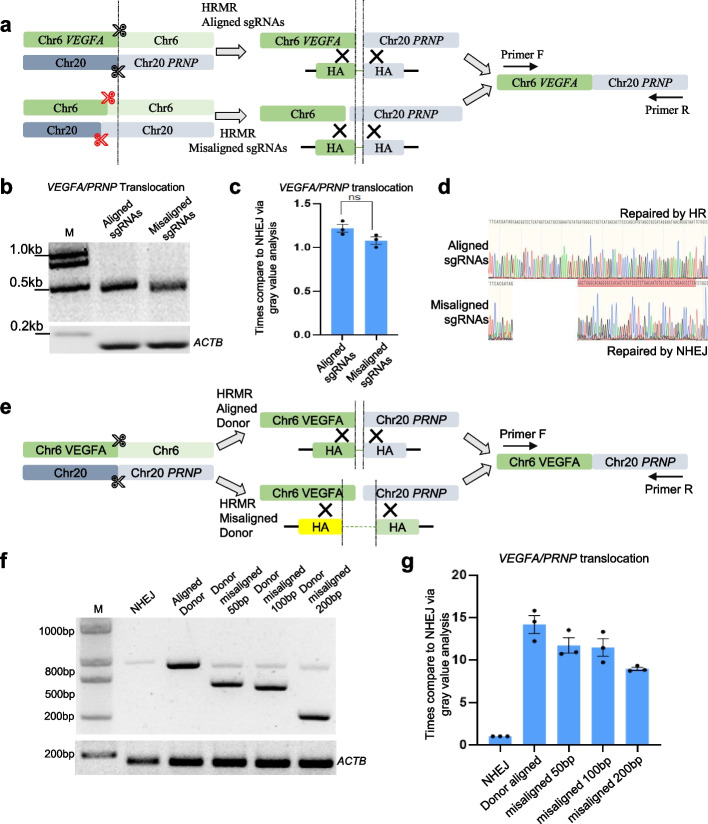
Misaligned sgRNAs and donors facilitate HRMR. **a** Schematic diagram of misaligned sgRNAs with homologous recombination-mediated chromosome rearrangement. Red scissors represent misaligned sgRNAs, and same HA donor was used. **b** The agarose gel image of aligned and misaligned sgRNAs at *VEGFA/PRNP* site by PCR analysis with primer flanking each side of translocation. The *ACTB* gene was amplified to ensure consistent genomic usage. **c** Relative translocation efficiency at *VEGFA/PRNP* site with aligned and misaligned sgRNAs determined via quantification of bands gray density in HEK293T cells. Independent biological replicates were performed (*n* = 3) and error bars show the s.e.m. **d** Sanger sequencing chromatograms showed different repair outcomes with aligned and misaligned sgRNAs. The red area represents a region different from the homologous donor. **e** Schematic diagram of misaligned homologous donor with homologous recombination-mediated chromosome rearrangement. **f** The agarose gel image of aligned and misaligned homologous donor at *VEGFA/PRNP* site by PCR analysis with primer flanking each side of translocation. **g** Relative translocation efficiency at *VEGFA/PRNP* site with aligned and misaligned homologous donor determined via quantification of bands gray density in HEK293T cells. Independent biological replicates were performed (*n* = 3) and error bars show the s.e.m

We next investigate whether misaligned sgRNAs or donors could still stimulate chromosome translocation. We designed sgRNAs targeting *VEGFA* and *PRNP* offset 60 bp upstream of the intended cut sites (Fig. 4a). Surprisingly, these misaligned guides improved efficiency similarly to the matched guides, though junction sequencing revealed repair occurred through NHEJ rather than HRMR (Fig. 4b–d). We also tested three donors with 50–200 bp homology mismatches from the cut sites (Fig. 4e). Misaligned donors enhanced efficiency but were less effective than the matched donors (Fig. 4f, g). Together, while perfectly matched sgRNAs and donors are optimal, HRMR retains partial activity even with substantial misalignments. The ability of mismatched donors to stimulate NHEJ also indicates they help bring together broken ends through homology.

### Live-cell tracking reveals dynamics of donor-mediated chromosomal repair

**Fig. 5:**
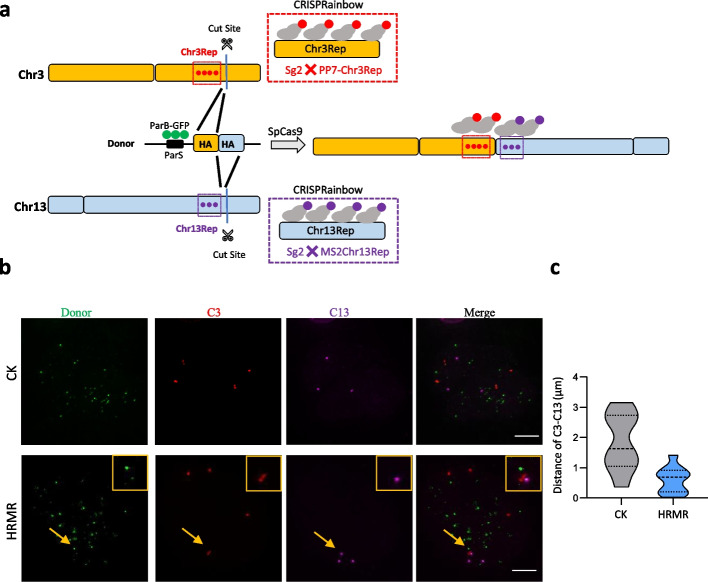
Living-cell images present homologous donor template bringing broken chromosomes ends into close proximity. **a** Diagram of donor induced translocation between Chr3 and Chr13 chromosomes. In U2OS cells, Chr3Rep and Chr3Rep were labeled with CRISPRainbow system. Donor plasmid was labeled by ParB-ParS system. SpCas9/sgRNA induces DSB between two marker sites by nuclear transfection (16 h after delivery of the DNA site marker system). **b** Fluorescence images of separation and reconnection of chromosome C3 and C13 sites. Red fluorescence represents the C3 site, and purple fluorescence represents the C13 site. Donor plasmid is shown by GFP fluorescence. HRMR, with homologous donor. CK, with non-homologous donor. The yellow boxes represent the DNA sites indicated by the arrows. Scale, 5 µm. **c** The distance between C3 and C13 sites was measured before and after chromosome reconnection in different cells

To observe the dynamics of chromosome rearrangement mediated by HRMR-mediated chromosome between Chr3Rep and Chr13Rep chromosomes, we utilized CRISPRainbow labeling approach for DSB ends in U2OS cells [8, 23–25]. CRISPRainbow enables real-time visualization of genomic loci by employing engineered sgRNAs with adaptor for fluorescent protein recruitment [23]. Specifically, we labeled the Chr3Rep and Chr13Rep loci with sg2XPP7-Chr3Rep/PCP-Halo and sg2XMS2-Chr13Rep/MCP-SNAP, respectively. These loci contain repetitive sequences, allowing robust fluorescent signal generation upon binding with ParB-GFP via the ParS sequence, providing a distinct green fluorescent signal to track the donor plasmid during recombination (Fig. 5a). Subsequently, 16 h after labeling, we introduced the sgRNA plasmids targeting the Chr3Rep and Chr13Rep adjacent genes to induce DSBs on these tagged genomic loci (Fig. 5a). This sequential delivery strategy facilitated easier labeling of the Chr3Rep and Chr13Rep sites before chromosome breaks. The anticipated outcome was a change in the localization pattern of the red and purple foci from isolation to co-localization following homologous recombination-mediated chromosomal rearrangement. As expected, when providing homologous donor, we captured the separation and fusion of Chr3Rep, Chr13Rep, and ParS within homologous donor, suggesting that homologous donor DNA increase spatial proximity between the DSB-containing chromosome domains. However, when providing non-homologous donor, Chr3Rep, Chr13Rep, and PasS within non-homologous donor were positioned at a distance from each other and no fusion of them was detected (Fig. 5b). Furthermore, we observed the co-localization of the Halotag marker of Chr3Rep gene and SNAP marker of the Chr13Rep gene (Fig. 5a, b), as the average distance decreased from 1.84 µm in cells with non-homologous donor to 0.661 µm in cells with homologous donor (Fig. 5c), which indicating that homologous donor has ability to recruit sequence with homology. In some cells, we also noted that the presence of green donor sites near the Chr3Rep and Chr13Rep sites, possibly suggesting the involvement of DNA damage or repair-related proteins and donor plasmids in promoting the proximity of the ends of the newly formed chromosomes after chromosome breaks for subsequent repair.

### Enhancing HRMR purity through inhibiting NHEJ pathway

**Fig. 6:**
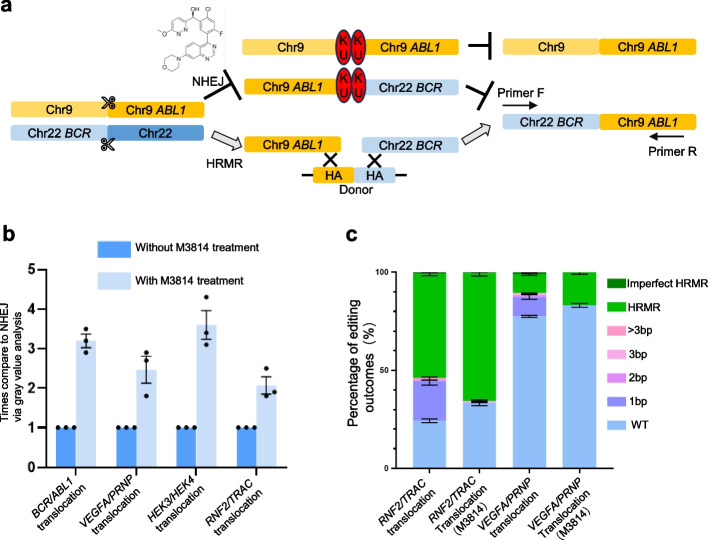
Inhibiting NHEJ pathway elevates the efficiency and purity of edit outcomes in chromosome rearrangement. **a** Schematic diagram of homologous recombination-mediated chromosome rearrangement with or without DNAPK inhibitor M3814. **b** Relative translocation efficiency at several sites with or without M3814 treatment determined via quantification of bands gray density. **c** HTS showed the percentage of editing outcomes with or without M3814 using primer insertion strategy. Different repair outcomes were marked as WT, indels including 1 bp, 2 bp, 3 bp, and > 3 bp, HRMR, and imperfect HRMR. Independent biological replicates were performed (*n* = 2) and error bars show the s.e.m. **d** Relative translocation efficiency at several sites with or without shRNA targeting KU70/80 and SCR7 determined via quantification of bands gray density. Independent biological replicates were performed (*n* = 3) and error bars show the s.e.m

DNA-dependent protein kinase catalytic subunit (DNA-PKcs) plays a pivotal role in the NHEJ pathway of DNA DSBs. Inhibiting DNA-PKcs’s enzymatic activity has been shown to transiently suppress NHEJ while enhancing homology-directed repair (HDR). We aimed to investigate whether temporarily inhibiting DNA-PKcs could facilitate HDR-mediated chromosomal translocations using the DNA-PKcs inhibitor M3814 (nedisertib) (Fig. 6a). Four distinct genomic loci were selected as potential sites of targeted chromosomal translocation. HEK293T cells were transfected with components to induce double-strand breaks and initiate HDR-mediated translocations in the presence or absence of M3814. HEK293T cells were pre-treated with 2 µM M3814 for 24 h prior to transfection. Twenty-four hours after transfection, the medium was replaced with fresh medium containing 2 µM M3814. After another 24 h, the medium was replaced again with fresh medium without M3814. Translocation frequencies were quantified using PCR quantifications. Treatment with M3814 resulted in a 2–fourfold increase in translocation efficiency across the four loci compared to untreated controls (Fig. 6b). The sequences flanking targeted breakpoints were amplified by PCR and subjected to Sanger sequencing to characterize translocation junctions. Cells treated with M3814 during both DSB induction and repair exhibited significantly fewer indels at translocation junctions than untreated controls (Fig. 6c). Together, these findings demonstrate that temporarily inhibiting DNA-PKcs with M3814 promotes high-fidelity chromosomal editing via HDR.

KU proteins and DNA ligase IV are also important proteins involved in NHEJ. To further validate that inhibiting proteins in the NHEJ pathway can enhance the efficiency of HRMR, we employed shRNA specifically targeting KU70/80 as well as the DNA ligase 4 inhibitor SCR7 pyrazine [26]. We attempted a chromosomal random translocation site (*VEGFA/PRNP*) and a pathological site (*BCR/ABL1*) for our experiments. Translocation frequencies were quantified using PCR quantifications. Treatment with Ku70/80 shRNA and SCR7 pyrazine resulted in a 2–fourfold increase in translocation efficiency across the two loci compared to untreated controls (Fig. 6d). These findings further support the conclusion that suppression of the NHEJ pathway promotes HRMR.

## Discussion

Precise and efficient genome editing at the chromosome scale possesses great therapeutic potential for diseases linked to chromosomal rearrangements. This study demonstrates that homologous donor can dramatically improve the efficiency of target chromosome rearrangements. Using HRMR in HEK293T cells, we observed substantial increases in the frequencies of both random translocations (e.g., *VEGFA/PRNP* and *RNF2/TRAC*) and pathological rearrangements (e.g., *BCR/ABL1* and *EML4/ALK*). These findings support the utility of HRMR for enabling biotechnological and clinical applications that manipulate whole chromosomes.

Upon DNA double-strand break induction, cells have several pathways available for repairing the chromosomal ends: error-prone repair mechanisms such as NHEJ and MMEJ, precise repair via HDR, or unrepaired DNA damage. Typically, cells tend to utilize the error-prone NHEJ pathway for double-strand DNA break repair. NHEJ repair necessitates the proximity of the two broken ends, which results in a lower efficiency of gene knock-ins compared to NHEJ-mediated repairs. However, the inherent physical distance between the ends of broken chromosomes hampers their interaction, leading to a relatively low probability of rearrangement. Chromosome imaging experiments have shown that homologous arms have the capacity to bring their matching sequences closer into closer proximity. This leads us to speculate that homologous recombination is not constrained by physical distance. Our observation likely explain that the occurrence of in situ chromosome dissociation following DSBs reduced the frequency of chromosomes rejoining, inhibiting NHEJ, and thereby promoting HDR in the presence of homologous donor or resulting in the absence of repair. We further hypothesize that drugs designed to inhibit NHEJ and enhance HDR efficiency may also improve the efficiency of HRMR. While the use of NHEJ pathway inhibitors can effectively enhance the efficiency and accuracy of HRMR at the tested loci, we speculate that for sites where classic NHEJ is infrequently involved, these inhibitors may have limited impact on HRMR efficiency [27].

The versatility of the HRMR strategy is highlighted by its ability to efficiently engineer precise chromosomal rearrangements across a diverse array of genomic loci and cell types. However, the fold increase in HRMR-mediated events (relative to NHEJ) does exhibit significant variability between different target sites. To investigate whether these differences may be associated with spatial chromatin positioning, we have analyzed available Hi-C data for several loci [28]. The results suggest that HRMR may be particularly advantageous for engineering rearrangements between loci with lower basal spatial correlation, as the homologous donors can bring distal chromosome ends into closer proximity to overcome the inherent physical separation that hinders NHEJ-mediated rejoining. However, there were exceptions, indicating that HRMR efficiency is influenced by multiple complex factors beyond just spatial chromatin organization, such as accessibility of the Cas9/gRNA complex, donor availability, and local chromatin environment. Nonetheless, the consistent superior performance of HRMR over conventional NHEJ-based approaches across a wide range of genomic targets underscores its broad utility for diverse applications in disease modeling, genome evolution studies, and therapeutic correction of pathogenic chromosomal aberrations.

## Conclusions

In summary, our study demonstrates the ability of homologous donors to significantly enhance chromosome rearrangement efficiency and provides insights into the underlying mechanisms driving this process. These findings highlight the potential of homologous recombination-mediated repair (HRMR) as a powerful tool for modeling and potentially treating diseases caused by chromosomal aberrations, such as Philadelphia chromosome-associated chronic myeloid leukemia and other cancers linked to chromosomal abnormalities.

## Methods

### Plasmid construction

To construct Cas9/gRNA plasmids, the parental Cas9 plasmid (Addgene plasmid #166,033) was digested by restriction enzymes XbaI and PciI to remove the standard sgRNA. The standard sgRNA was replaced with designed sgRNAs by Golden Gate cloning. The sgRNA inserts were flanked by BsaI restriction sites to allow cloning of other additional sgRNAs. All sgRNA sequences used in this study are listed in Additional file 2: Supplementary Table S1. To construct Cas9-MMLV plasmids, the parent nCas9-MMLV plasmid was digested by restriction enzymes SalI and NotI to remove the nCas9, and then inserted original Cas9.

To construct the homologous donor plasmids, HAs corresponding to all loci were designed by using the human genomic DNA sequence from NCBI. Homologous donor sequences are listed in Additional file 2: Supplementary Table S3. HAs were PCR amplified from human embryonic kidney (HEK) 293 T genomic DNA by using either Q5 High-Fidelity DNA Polymerase (M0494S, New England Biolabs) or KOD DNA Polymerase (KOD-101, Toyobo). HA inserts were cloned into KpnI and BamHI restriction sites of pCDH. To construct the NIS-homologous donor plasmids, NIS sequences were either obtained from the parental Cas9 plasmid or were synthesized (GeneWiz). NIS sequences were cloned into the EcoRI and NheI restriction sites at the 3′ ends of HAs.

To construct the plasmids for the living-cell imaging, P2A-BsParB-sfGFP was inserted into EcoRI and BamHI restriction sites of the parental Cas9 plasmid, ensuring in-frame fusion with Cas9. Chr3rep sgRNA-2xMS2 and Chr13rep sgRNA-2xPP7 were cloned to AgeI and XbaI restriction sites of the sgRNA plasmid under independent U6 promoter. MCP-Halo and PCP-SNAP were cloned to NheI and EcoRI restriction sites of expression plasmid under independent EF1S promoter. The templates for MCP-Halo and PCP-SNAP were synthesized (GeneWiz) based on sequences from Addgene plasmids (#121,937 and #75,386) as described previously [23].

### Cell cultures and transfection

HEK293T, Hela, and U2OS cells were cultured in Dulbecco’s modified Eagle’s medium (DMEM; 11,995,065, Gibco) supplemented with 10% fetal bovine serum (FBS; 10,100,147, Gibco), 100 U/ml penicillin, and 100 μg/ml streptomycin (60162ES76, Yeasen) at 37 °C with 5% CO_2_. Cells were seeded into 24-well plates (~ 4–5 × 10^5^ cells/well). Plasmids were extracted by using the Endo-Free Mini Plasmid Kit (DP118-02, Tiangen) before transient transfection by using EZ trans (AC04L091, Life-ilab Biotech) following the manufacturer’s protocols. For each well, 350 ng of Cas9 plasmid (or the associated plasmid), 200 ng of dual sgRNAs plasmid, and 200 ng of homologous donor plasmid were used in HRMR group and 200 ng of control plasmid was used in NHEJ group. The medium was replaced 12–18 h after transfection, and cells were harvested for flow cytometric analysis and microscopy at 72 h post-transfection. For live-cell imaging experiments, U2OS cells were seeded into 6 mm dish. For each dish, 500 ng of Cas9-P2A-BsParB-sfGFP plasmid, 1000 ng of chr3rep and chr13rep plasmid, 200 ng of MCP-Halo and PCP-SNAP plasmid, and 200 ng of homologous or non-homologous donor with 8xParS. Sixteen hours later, 500 ng of dual sgRNAs plasmid for cutting chr3 and chr13 was transfected into U2OS cells. For WT-PE constructs, cells were transfected with 350 ng Cas9-MMLV plasmid, 250 ng sgRNA expression plasmid, and 3 µl of EZ trans (AC04L091, Life-ilab Biotech). For Ku70/80 protein knock down experiments, HEK293T cells were seeded into 24-wells plate. For each well, 350 ng of Cas9 plasmid, 150 ng of dual sgRNAs plasmid, 200 ng of homologous donor plasmid, and 150 ng of Ku70/80 shRNA plasmid were used in Ku knock-down group and 150 ng of control plasmid was used in control group. All the cell lines tested negative for mycoplasma.

### Flow cytometry analysis

For plasmid translocation experiment, at least 10,000 cells were analyzed by using either a BD LSR Fortessa flow cytometer (BD Biosciences) or a Beckman Coulter CytoFLEXS (Beckman Coulter Life Sciences). Either a BD FACS AriaIII or aBD FACS Aria Fusion (BD Biosciences) were used to sort gated cells. Cells that harbored the GFP and BFP were directly sorted into 1.5 ml tube for DNA extraction. Batch processing of fluorescence-activated cell sorting (FACS) data was performed and analyzed by using FlowJo software, version 10.

### Genomic PCR

Genomic DNA was extracted from cells by using Quick Extract DNA extraction solution 1.0 (QE0905T, Lucigen) or Genomic DNA Extraction Kit (DP304, Tiangen) following the manufacturers’ protocols. Briefly, cells were harvested 72 h after transfection and washed with phosphate-buffered saline (PBS) three times. Lysis buffer was added to the cells, and samples were incubated at 65 °C for 6 min and then at 98 °C for 2 min. Genomic polymerase chain reaction (PCR) was usually performed in a mixture containing Ex Taq DNA polymerase (RR006Q, Takara), 0.5 μl of 4 µM duplex DNA substrate (400 nM final), 10 pmol (0.2 μM) primers, 0.5 mM dNTP mix, 20 mM HEPES-K, pH 7.5, 100 mM KCl, 5% glycerol, 0.2 mM EDTA, pH 8.0, 3 mM MgCl2, and 5 mM dithiothreitol (DTT). PCRs were performed as follows: 95 °C for 3 min, 28 cycles of (95 °C for 30 s, 60 °C for 30 s, and 72 °C for 30 s) followed by a final extension at 72 °C for 5 min. PCR primers are listed in Additional file 2: Supplementary Table S3. PCR products were analyzed by Sanger sequencing (GeneWiz).

### Analysis of chromosome translocation frequencies of PCR

Common PCR was conducted to amplify the targeted region from extracted genomic DNA by using primers flanking the HAs. Junction PCR was conducted to amplify the junction region by using site-specific primers. Wild-type and truncated genomic fragments were resolved by gel electrophoresis. All PCR primer sequences are listed in Additional file 2: Supplementary Table S2.

### Next-generation sequencing library preparation

At 72 h after transfection, genomic DNA was extracted using a DNA Extraction Kit (Tiangen). Total DNA (250 ng) was used for next-generation sequencing (NGS) library preparation. *VEGFA/PRNP* translocation and *RNF2/TRAC* were amplified using specific primers in a first-round PCR. For each sample, > 50 ng of purified PCR fragment was used for library preparation. PCRs (50 μl) contained 0.5 μM of each forward and reverse primer, 1 μl of genomic DNA extract, and 25 μl of PrimeSTAR® HS Premix (R040Q, Takara). PCRs were carried out as follows: 98 °C for 2 min, 28 cycles of (98 °C for 10 s, 61 °C for 30 s, and 72 °C for 30 s), followed by a final 72 °C extension for 2 min. PCR products were treated in a single reaction with End Prep Enzyme Mix to repair ends, to phosphorylate 5′ ends, and to add dA tails to 3′ ends. Then, T-A ligation was performed to add adapters to both ends. Adapter-ligated DNA was purified using DNA Clean Beads (A63882, Beckman Coulter). A second PCR was performed with P5 and P7 primers carrying sequences that anneal with flow cells (for bridge PCR) and indexes (for multiplexing). Specifically, PCRs (25 μl) contained 0.5 μM of each unique forward and reverse Illumina barcoding primer pair (I7/I5), 1 µl of purified adapter-ligated DNA, and 12.5 μl of PrimeSTAR® HS Premix. The PCRs were carried out as follows: 98 °C for 2 min, 10 cycles of (98 °C for 10 s, 61 °C for 30 s, and 72 °C for 30 s), followed by a final 72 °C extension for 2 min. The final library product for sequencing was then purified by beads and qualified. The qualified libraries were pair-end sequenced (300 bp) on the Illumina MiSeq System.

### High-throughput sequencing data analysis

Alignment of amplicon sequences to a reference sequence was performed using CRISPResso2 [29]. The quantification window was increased to 10 bp around the expected cut site to better capture diverse editing outcomes. Only reads containing no mismatches to the expected amplicon were considered for correct editing; reads containing indels that differed from the expected amplicons and reference sequence were included in error editing.

### Living-cell imaging

All living-cell imaging was carried out on a Delta Vision Ultra imaging system (GE Healthcare). The U2OS cells were cultured on No. 1.0 glass bottom dishes (MatTek). The microscope stage incubation chamber was maintained at 37 °C and 5% CO_2_. GFP was excited at 488 nm and collected using filter at 498/30 nm (wavelength/bandwidth); HaloTag-JF549 was excited at 561 nm, and its emission was collected using filter at 609/37 nm (wavelength/ bandwidth); SNAP-JF647 was excited at 647 nm, and its emission was collected using 661/25 nm. Imaging data were acquired by DeltaVision imaging (GE Healthcare Inc.) software. For the representative images, the raw data were deconvoluted by softWoRx (GE Healthcare Inc.) software.

### Statistical analysis

All statistical data are shown as means ± SEM of at least three replicates using GraphPad Prism (San Diego, CA, USA, version9.3.1). Two-tailed Student’s *t*-test was used to determine the *P* value between two groups. For all figures, *, **, and *** indicate *P* < 0.05, *P* < 0.01, and *P* < 0.001, respectively, and ns indicates no significance.

## Supplementary Information

Additional file 1: Fig. S1. Homologous donor also promotes dsDNA recombination. Fig. S2. Determination of random rearrangement and pathological rearrangement sites between NHEJ and HRMR in HEK293T cells. Fig. S3. Sanger sequencing chromatograms showed different repair outcome when using NHEJand HRMRat different endogenous loci. Fig. S4. Optimization of the length of homologous arm and insertion of HRMR. Fig. S5. Different donors promote different forms of chromosome rearrangement. Fig. S6. I-SceI enzyme digestion to determine the relative proportion of HR and NHEJ occurrence. Fig. S7. Detailed explanation of the primer insertion method. Fig. S8. Sequence alignment of VEGFA sites edited by HRMR using primer insertion strategy.

Additional file 2: Supplementary Table S1. List of sgRNA sequences. Supplementary Table S2. List of primer sequence used in this study. Supplementary Table S3 Representative homologous arm sequence.

Additional file 3. Uncropped DNA gel electrophoresis images.

## Data Availability

All data supporting the findings of this study are available in the article or in the supplementary information files. Sequencing raw data was deposited to SRA BioProject (accession number PRJNA1229868) [30]. Microscopy data was deposited to Figshare (accession number 28456466) [31].
