# Supplementary material for: Recruitment and rejoining of remote double-strand DNA breaks for enhanced and precise chromosome editing
Source: Genome Biol. 2025 Mar 11;26:53. doi: 10.1186/s13059-025-03523-8 (PMC11895233; doi:10.1186/s13059-025-03523-8)
Supplement: Supplementary file 1 — Additional file 1: Fig. S1. Homologous donor also promotes dsDNA recombination. Fig. S2. Determination of random rearrangement and pathological rearrangement sites between NHEJ and HRMR in HEK293T cells. Fig. S3. Sanger sequencing chromatograms showed different repair outcome when using NHEJand HRMRat different endogenous loci. Fig. S4. Optimization of the length of homologous arm and insertion of HRMR. Fig. S5. Different donors promote different forms of chromosome rearrangement. Fig. S6. I-SceI enzyme digestion to determine the relative proportion of HR and NHEJ occurrence. Fig. S7. Detailed explanation of the primer insertion method. Fig. S8. Sequence alignment of VEGFA sites edited by HRMR using primer insertion strategy. [file 13059_2025_3523_MOESM1_ESM.pdf]

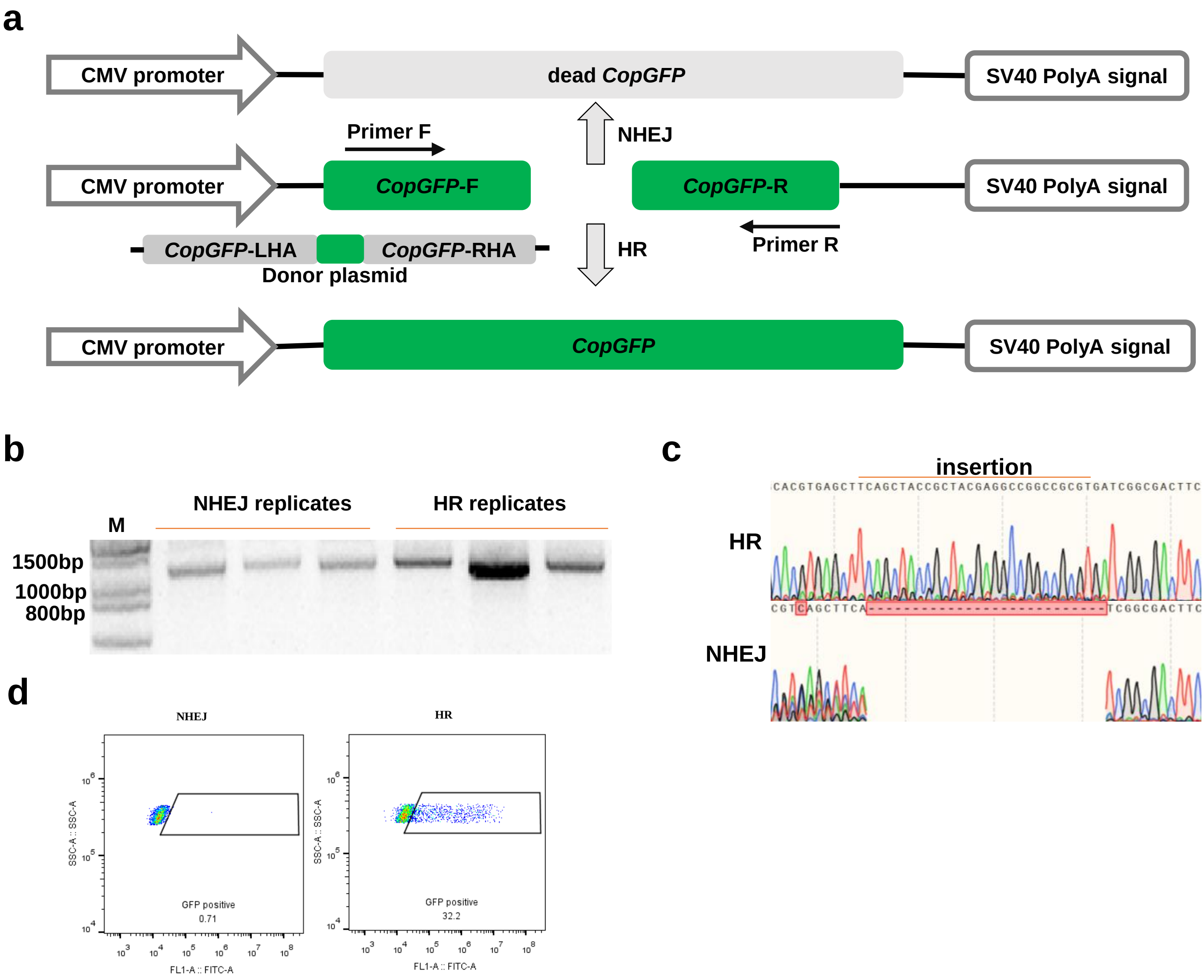

Figure S1 . Homologous donor also promote dsDNA recombination.

a, Schematic diagram of dsDNA rearrangement. Occurrence of NHEJ does not enable the correct ligation between CopGFP fragments while introduction of homologous arms enables the correct ligation of CopGFP to produce active CopGFP.

b, Gel electrophoresis showed that the introduction of homologous arms had a positive effect on the rearrangement of dsDNA.

c, Sanger sequencing shows homology recombination was major repair way.

d, The FACS diagram shows correct ligation of CopGFP fragments only appears when adding homologous donor.

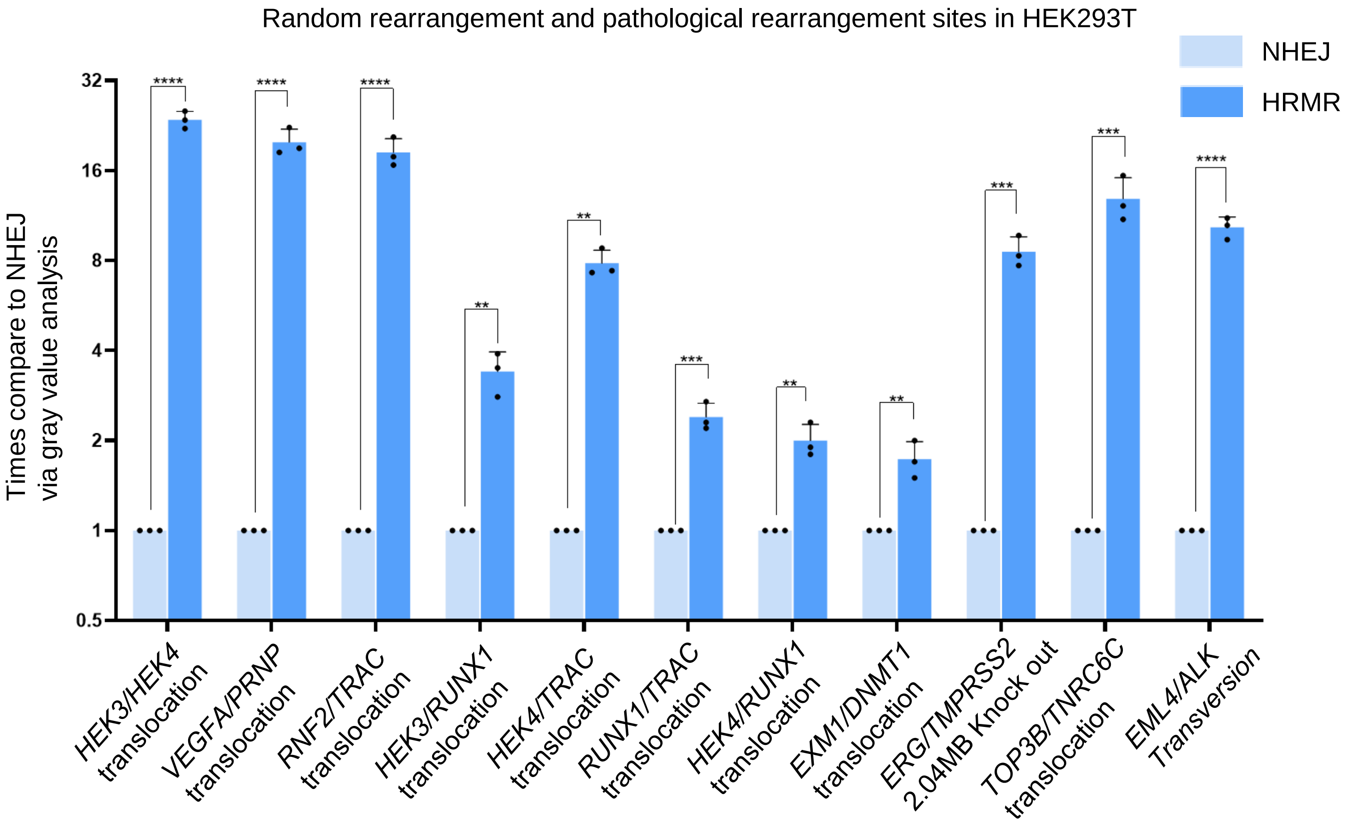

Figure S2 . The versatility of HRMR across various genomic sites encompassing both random and pathological chromosome translocation.

Determination of random rearrangement and pathological rearrangement sites between NHEJ and HRMR in HEK293T cells via quantification of bands grey density. Independent biological replicates were performed ($n = 3$) and error bars show the s.d.

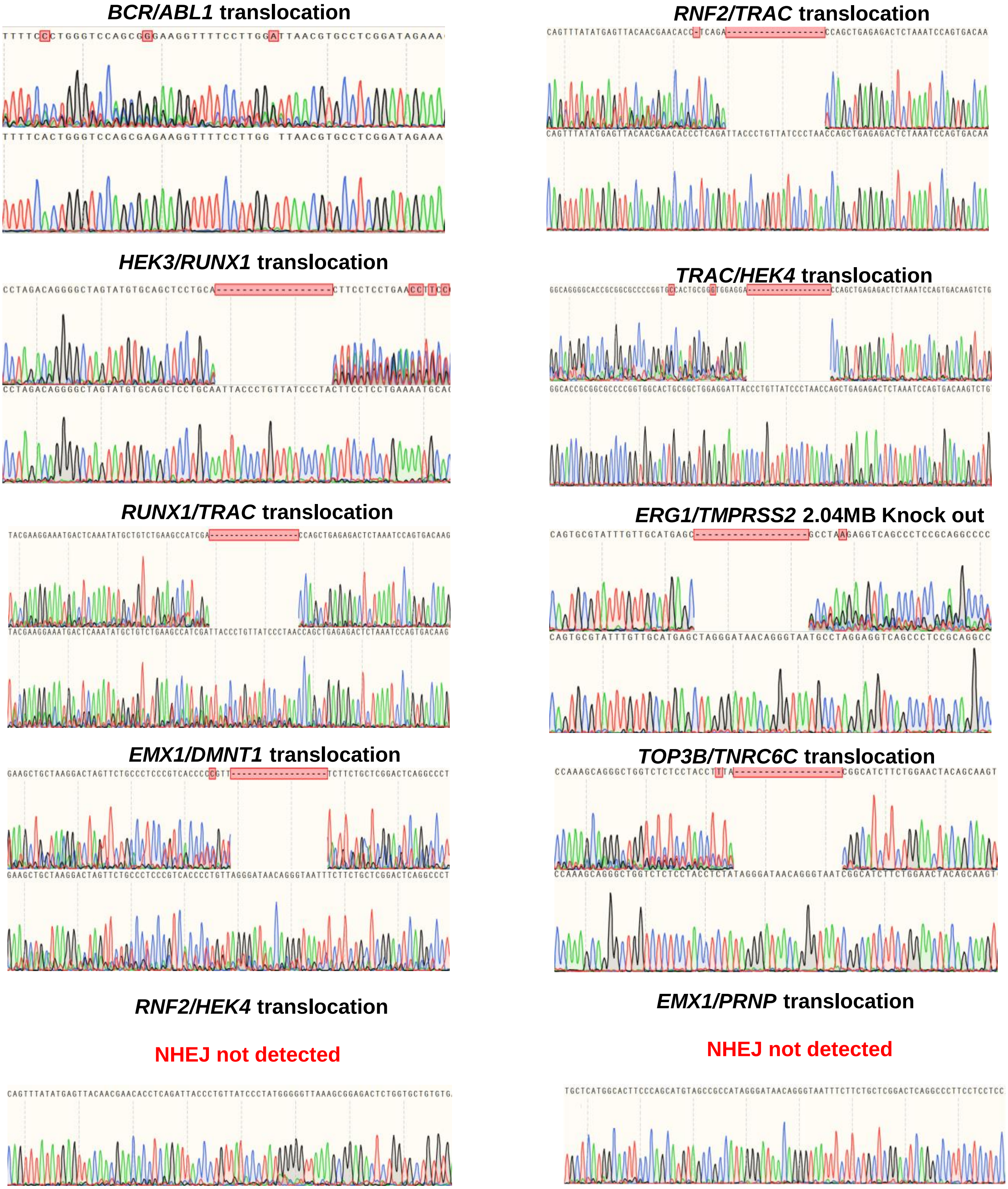

Figure S3. Sanger sequencing shows HRMR is a precise pathway for engineering chromosomal rearrangements.
Sanger sequencing chromatograms showed different repair outcome when using NHEJ（upper panel） and HRMR（lower pannel） at different Endogenous loci. Notably, no translocation events of *RNF2/HEK4* translocation and *EMX1/PRNP* were detected because of failure translocation events amplified.

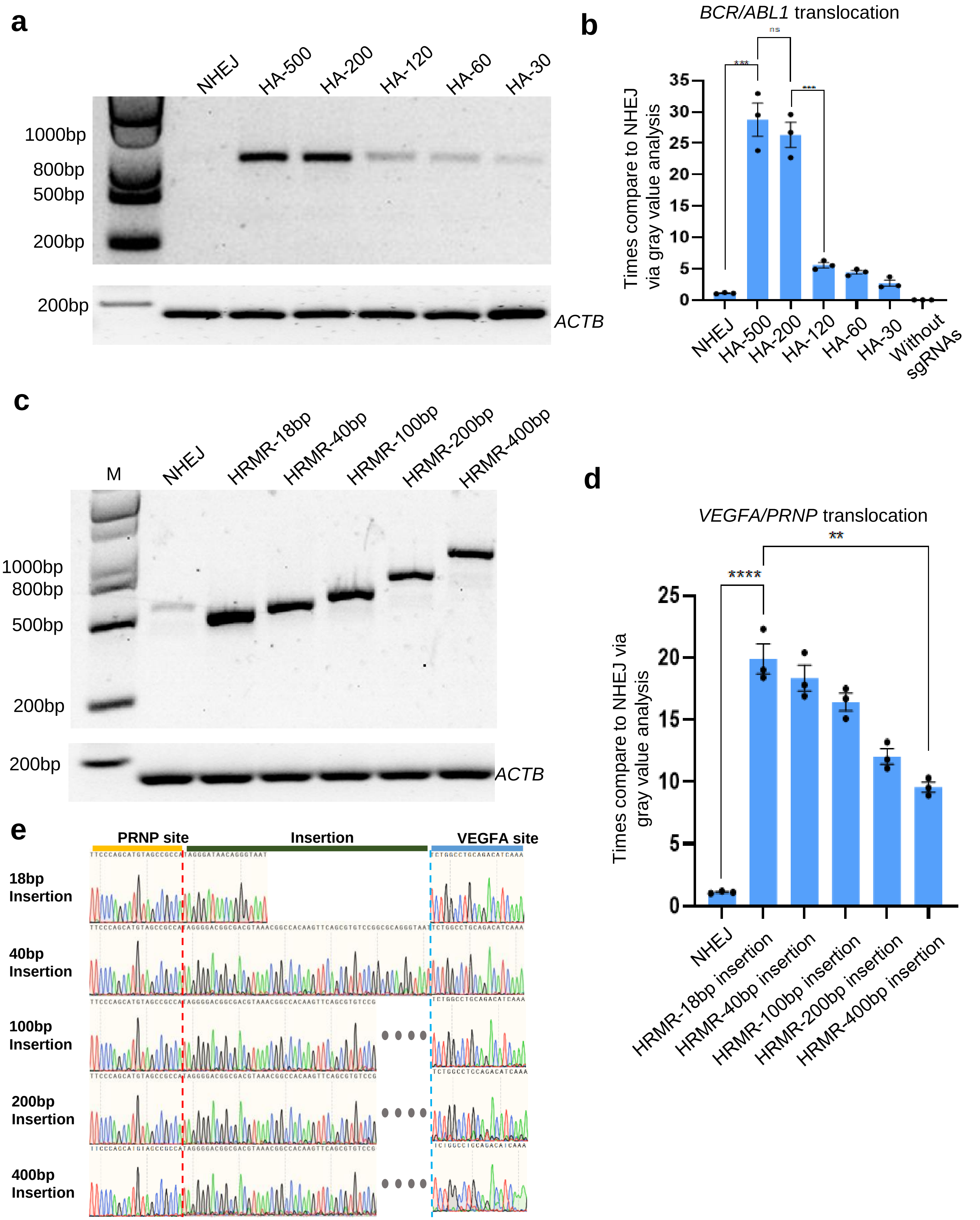

Figure S4 . Optimization of the length of homologous arm and insertion of HRMR.

a,**b**, Agarose gel analysis of different homologous arm lengths promoting chromosome rearrangement, and quantifying the target rearrangement through image J analysis of band grayscale. Independent biological replicates were performed (*n* = 3) and error bars show the s.e.m.

c,**d**, Agarose gel analysis of different insertion lengths promoting chromosome rearrangement, and quantifying the target rearrangement through image J analysis of band grayscale. Independent biological replicates were performed (*n* = 3) and error bars show the s.e.m.

e, Sanger sequencing chromatograms showed that different insertion length are integrated into chromosome translocation sites.

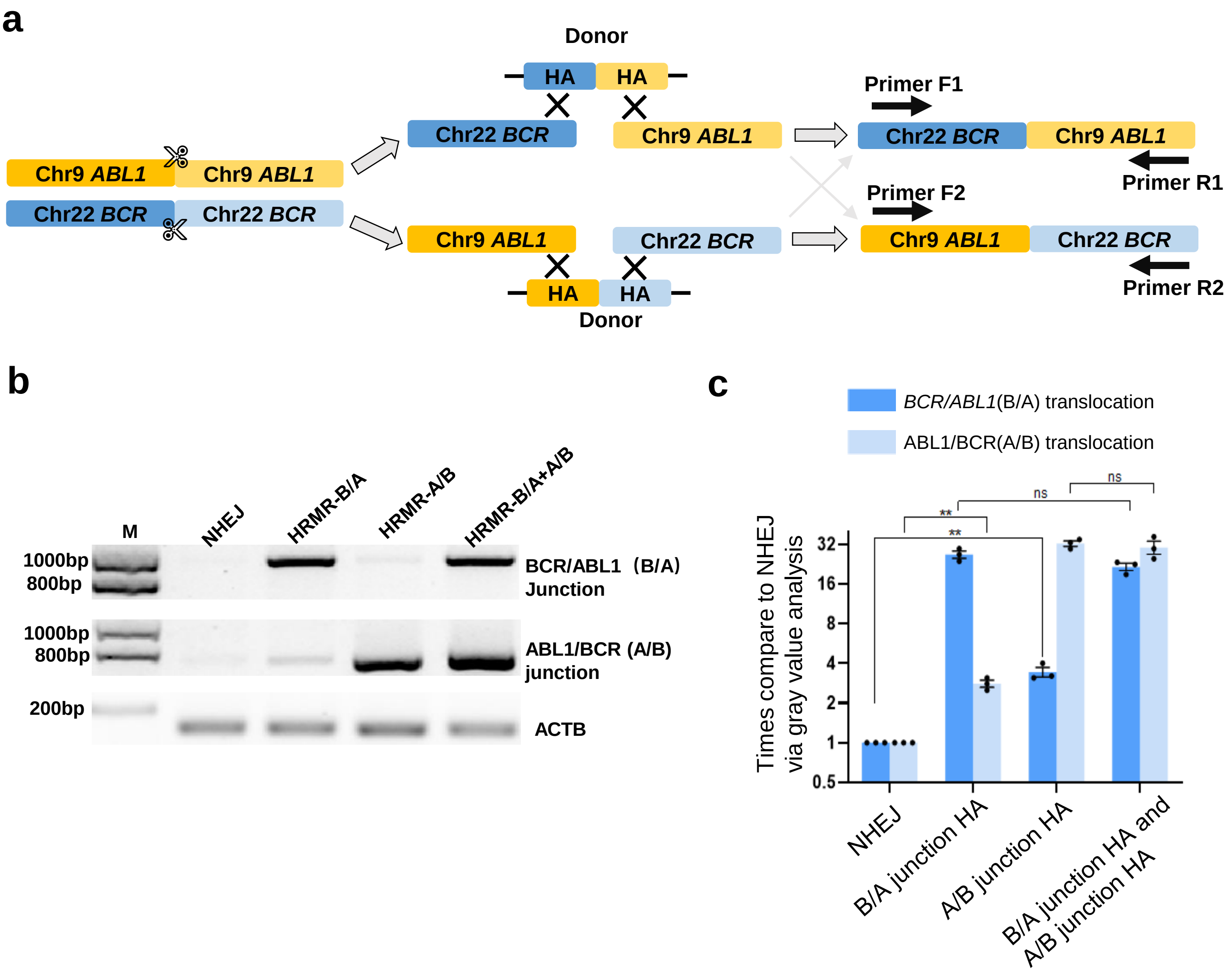

Figure S5 . Different donors promote different forms of chromosome rearrangement.

a, Schematic diagram for homologous arm mediated translocation, designing a pair of sgRNAs targeting the target gene. Using different donors for different chromosome rearrangement.

b，c， Agarose gel analysis of different homologous donors promoting different forms of chromosome rearrangement, and quantifying the target rearrangement through image J analysis of band grayscale. Independent biological replicates were performed ($n = 3$) and error bars show the s.e.m.

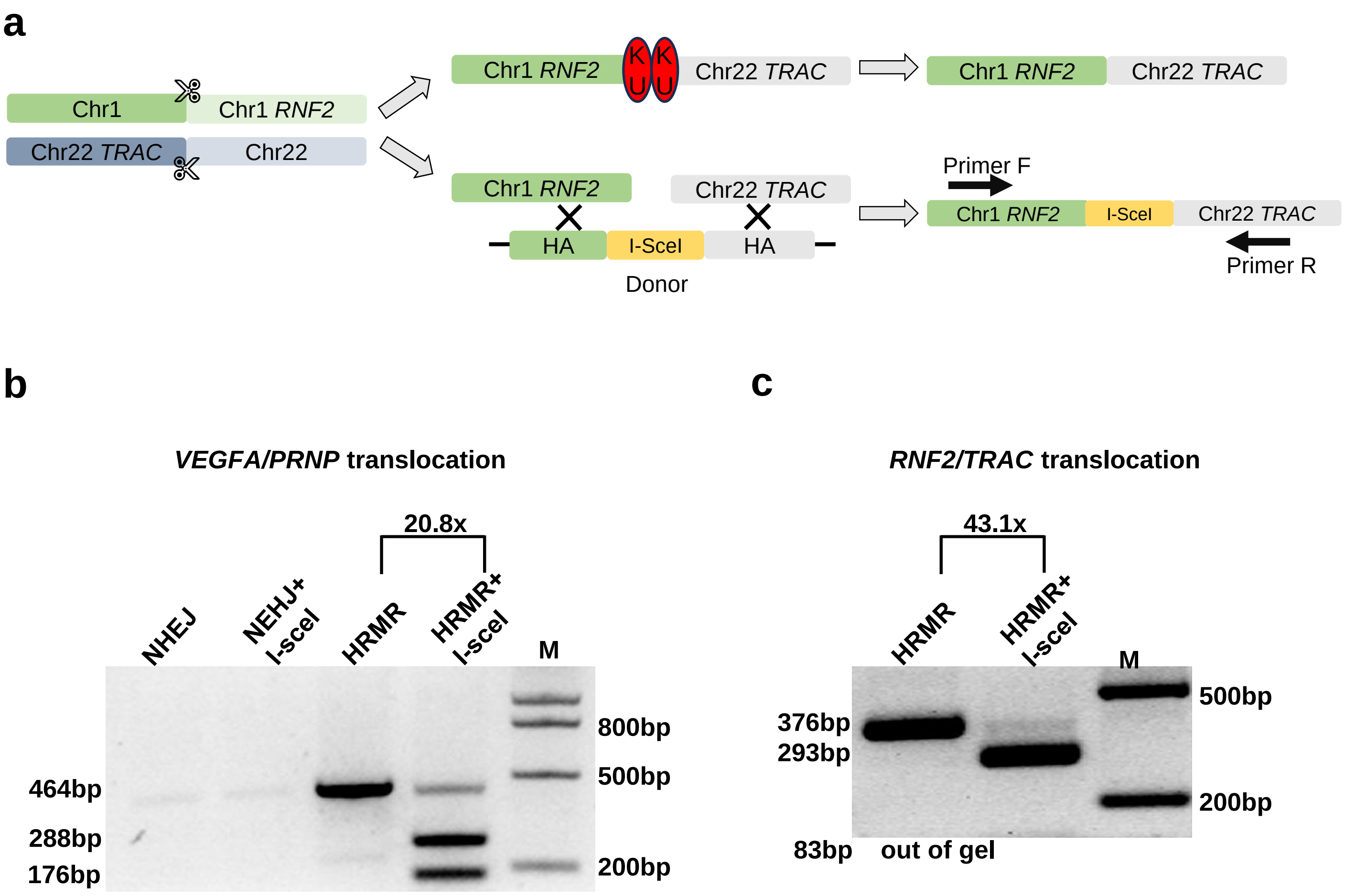

Figure S6 . I-SceI enzyme digestion to determine the relative proportion of HR and NHEJ occurrence.
a, Schematic diagram for homologous arm mediated translocation, designing a pair of sgRNAs targeting the target gene. KU protein mediated NHEJ and homologous recombination mediated by homology donor. A pair of primers was used to detect rearranged chromosomes.
b, **c**, Agarose gel analysis of I-SceI digestion of targeted translocation amplifiers. The bands with size match sequences with or without enzyme digestion were indicated, and the multiples displayed by grayscale analysis are displayed above the gel plot.

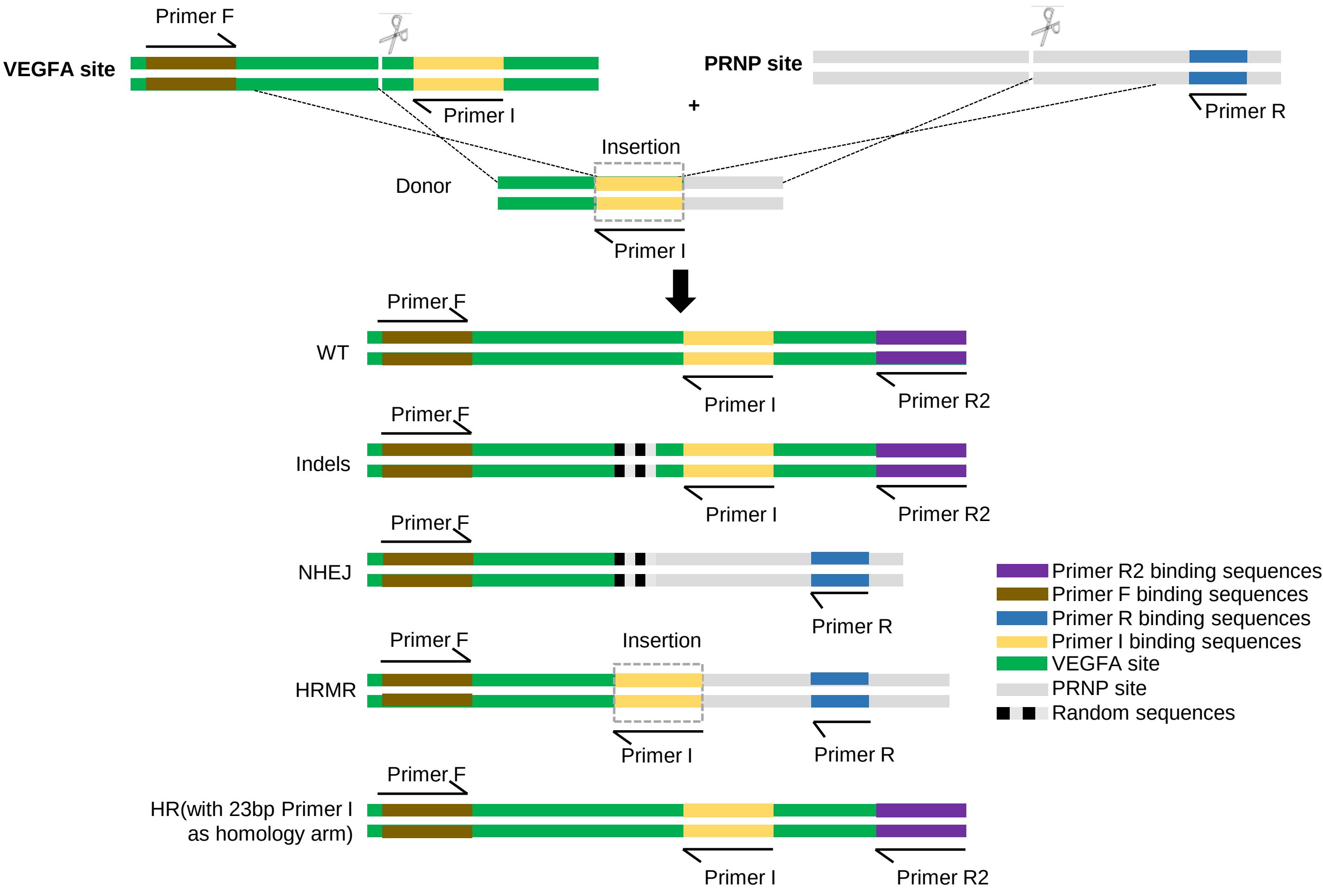

Figure S7. Detailed explanation of the primer insertion method.
Schematic diagram of primer insertion to quantifying the efficiency and accuracy of HRMR-mediated chromosome rearrangement.
1. Wild-Type VEGFA (WT): The VEGFA locus remains unmodified or is repaired without indel formation. Both Primer F and Primer I can amplify this sequence.
2. Indels: Following Cas9-induced cleavage at the VEGFA locus, a double-strand break is repaired through NHEJ pathway, leading to indels (small insertions or deletions). Despite the presence of indels, both Primer F and Primer I can still bind to the genome and amplify the sequence.
3. NHEJ-mediated translocations: Double-strand breaks (DSBs) at VEGFA and PRNP are repaired via the NHEJ pathway, resulting in rearranged chromosomes without the insertion of Primer I (with or without indels). In this case, Primer F cannot amplify the product in combination with Primer I. However, the NHEJ-mediated translocation can still be detected using Primer F and Primer R.
4. HRMR-mediated translocations: Homology-directed repair (HDR) using the homologous donor results in translocations that include the insertion of Primer I. This specific product can be amplified using Primer F and Primer I. Additionally, Primer F and Primer R can be used to detect the products of HRMR-mediated translocations.
5. HR (with 23bp Primer I as homology arm)：Primer I exhibits homology to the VEGFA genome, Primer I might function as a homologous arm to mediate HR events. This specific product can be amplified using Primer F and Primer R2.
Primer F and Primer I were used to simultaneously amplify three products: wild-type (WT), indels, HRMR-mediated translocations and rare HR (with 23bp Primer I as homology arm). The proportions of these products were then determined through sequence analysis. Additionally, Primers F and Primer R were used to amplify two products: NHEJ-mediated translocations and HRMR-mediated translocations, allowing further proportion analysis. Primers F and Primer R2 were used to amplify three products：wild-type (WT), indels, and HR (with 23bp Primer I as homology arm) events.

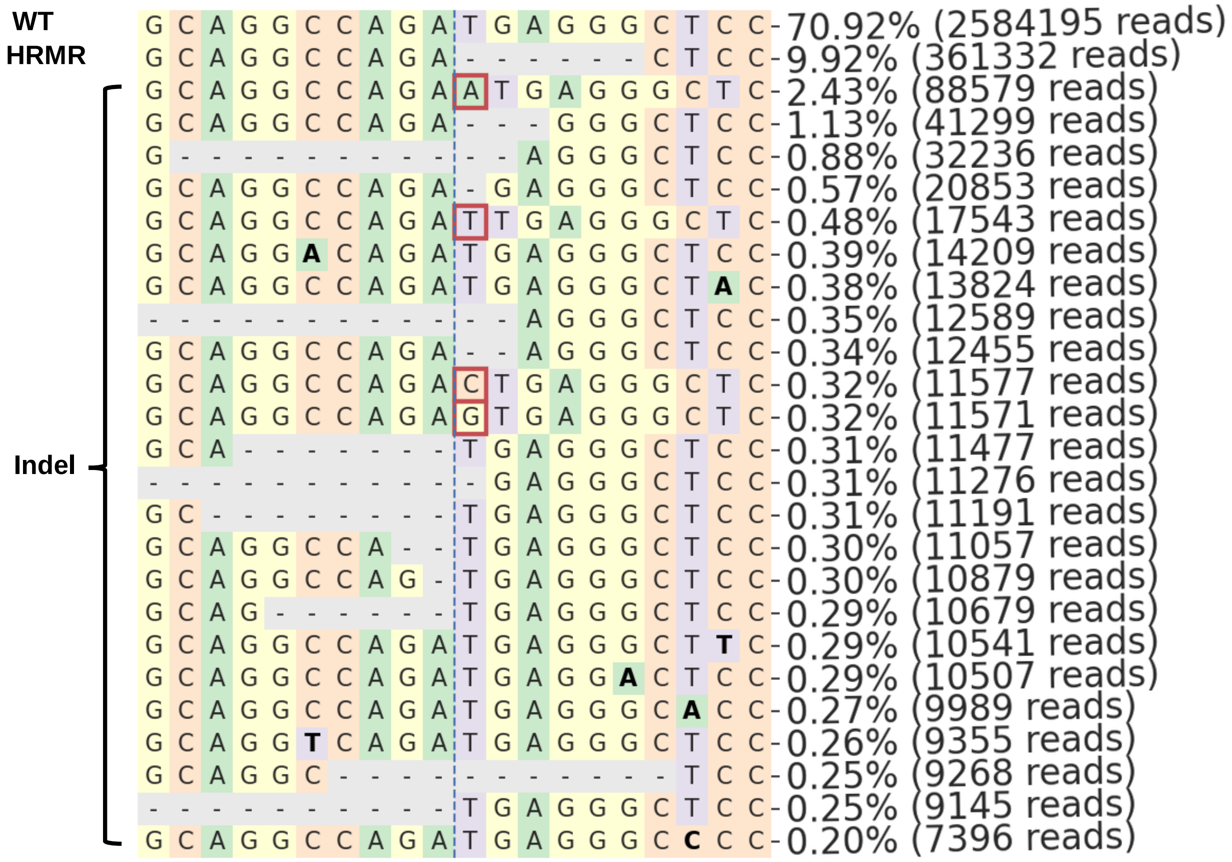

Figure S8 . Sequence alignment of *VEGFA* sites edited by HRMR using primer insertion strategy (*VEGFA/PRNP* translocation). Wildtype *VEGFA* sequence was used as a reference and HTS sequencing reads were aligned to the reference sequence and top sequences with a ratio over 0.2% were shown. Vertical dashed line marked the position of Cas9 induced DSBs. Red box marked the inserted bases and the short-term marked the deleted bases. Accurate six base deletion was considered as HRMR.
