# Supplementary material for: Recruitment and rejoining of remote double-strand DNA breaks for enhanced and precise chromosome editing
Source: Genome Biol. 2025 Mar 11;26:53. doi: 10.1186/s13059-025-03523-8 (PMC11895233; doi:10.1186/s13059-025-03523-8)
Supplement: Supplementary file 2 — Additional file 2: Supplementary Table S1. List of sgRNA sequences. Supplementary Table S2. List of primer sequence used in this study. Supplementary Table S3 Representative homologous arm sequence. [file 13059_2025_3523_MOESM2_ESM.pdf]

Additional file 2：Supplementary Table S1. List of sgRNA sequences used in this study.

sgRNA	targeted sequence (5' - 3')	Reference
VEGFA	GATGTCTGCAGGCCAGATGA	15
PRNP	GCAGTGGTGGGGGGCCTTGG	15
HEK3	GTCAACCAGTATCCCGGTGC	15
HEK4	GGCACTGCGGCTGGAGGTGG	15
RNF2	GTCATCTTAGTCATTACCTG	15
TRAC	AGAGTCTCTCAGCTGGTACA	15
EMX1	GAGTCCGAGCAGAAGAAGAA	15
RUNX1	GCATTTTCAGGAGGAAGCGA	15
BCR	TATCCGAGGCACGTTAAGGG	10
ABL1	AGCCGCTCGTTGGAACTCCA	21
DNMT1	GATTCCTGGTGCCAGAAACA	15
ERG1	GTATTTGTTGCATGAGCTCC	14
TMPRSS2	GGGCTGACCTCCTAGGCCAT	14
TOP3B	GGTCTCTCCTACCTCTAGAG	14
TNRC6C	AGTTCCAGAAGATGCCGCTT	14
EML4	GACCTGAACAGCAAGTTTGT	6
ALK	GGCCTTGCTGAAACTTCCTT	6
chr11 16.8MB Knock out-HBB	CACGTTCACCTTGCCCCACA	7
chr11 16.8MB Knock out-FANCH	GGGGTCCCAGGTGCTGACGT	7
VEGFA-misaligned sgRNA	ATGTACAGAGAGCCCAGGGC	15
PRNP-misaligned sgRNA	GCATGTTTTCACGATAGTAA	15
chr3 imaging sgRNA	TGATATCACAG	23
chr13 imaging sgRNA	ACCATTCCTTC	23
SPACA7	GGATGTAGGAGTCAGTCCAG	This study
PPP1R2	GTTAGCTGACTTGAAGAAAA	This study
chr11-HBB-pegRNA-I-secI insertion	CACGTTCACCTTGCCCCACAGT TTTAGAGCTAGAAATAGCAAGTT AAAATAAGGCTAGTCCGTTATCAA CTTGAAAAAGTGGCACCGAGTCG GTGCATTACCCTGTTATCCCTAGGG GCAAGGTGAAC	7
chr11-FANCH-pegRNA-I-secI insertion	GGAATCCCTTCTGCAGCACCGTTTT AGAGCTAGAAATAGCAAGTTAAAAT AAGGCTAGTCCGTTATCAACTTGAAA AAGTGGCACCGAGTCGGTGCTAGGG ATAACAGGGTAATGCTGCAGAAGGGAT	7

Additional file 2：Supplementary Table S2. List of primer sequence used in this study.

Primer name	Sequence (5' - 3')
BCR/ABL1-BCR-F	GGGCTGGTTGCTTCACAAAG
BCR/ABL1-ABL1-R	CATTCCCCATTGTGATTATAGCCTAAGACC
ABL1/BCR-BCR-F	CCAAACTAAGAGATGGGCAGAACTG
ABL1/BCR-ABL1-R	CCCTTCAGCGGCCAGTAGCA
VEGFA/PRNP-VEGFA-F	AGGAACAAGGGCCTCTGTCT
VEGFA/PRNP-PRNP-R	TTGTGATATTGACGCAGTCGTGC
HEK3/HEK4-HEK3-F	ACGCCTGTGATGGGCTAATTG
HEK3/HEK4-HEK4-R	TTCAACCCGAACGGAGACAC
RNF2/TRAC-RNF2-F	TGCAGACAAACGGAACTCAAC
RNF2/TRAC-TRAC-R	AAGGAAACAGCCTGCGAAGG
Chr11 KO-HBB-F	CACGTTGCCCAGGAGCTGTG
Chr11 KO-FANCF-R	GCAGAGAGTCGCCGTCTCCA
HEK3/RUNX1-HEK3-F	ACGCCTGTGATGGGCTAATTG
HEK3/RUNX1-RUNX1-R	ATCACCAACCCACAGCCAAG
HEK4/TRAC-HEK4-F	CCAGTGGTTCAATGGTCATCCCA
HEK4/TRAC-TRAC-R	AAGGAAACAGCCTGCGAAGG
RUNX1/TRAC-RUNX1-F	GACAAAGTTCTCACGCACCGAC
RUNX1/TRAC-TRAC-R	AATGGATAAGGCCGAGACCAC
HEK4/RUNX1-HEK4-F	CCAGTGGTTCAATGGTCATCCCA
HEK4/RUNX1-RUNX1-R	GAATGCAAACCACAGGGTTTCG
EMX1/DNMT1-EMX1-F	GGGGCCCCTAACCCTATGTA
EMX1/DNMT1-DNMT1-R	GGGACCGTTTGAGGAGTGTT
ERG/TMPRSS2-ERG-F	GGCAGCTTGAGACACTGGT
ERG/TMPRSS2-TMPRSS2-R	GCAGTATGACATGGCCAGAGT
TOP3B/TNRC6C-TOP3B-F	CCTGTAACACATAAACGACCGTG
TOP3B/TNRC6C-TNRC6C-R	CTCCAAGCATGGCCACTCT
EML4/ALK-EML4-F	AGCTCTGAACCTTTCCATCATACT
EML4/ALK-ALK-R	GTGTCCTCCCTCTCGTGGTAA
VEGFA/PRNP-primer insertion-VEGFA-F	AGGAACAAGGGCCTCTGTCT
VEGFA/PRNP-primer insertion-primerV1	CTCTGACAATGTGCCATCTGGAG
RNF2/TRAC-primer insertion-primerT1	CCCACAGATATCCAGAACCCTG
RNF2/TRAC-primer insertion-TRAC-R	AAGGAAACAGCCTGCGAAGG
VEGFA-misaligned donor-VEGFA-F	GTCTGGGCTTGGGCTGATAGAA
VEGFA-misaligned donor-PRNP-R	TCATCTTAACGTCGGTCTCGGT

Additional file 2：Supplementary Table S3. Representative homologous arm sequence.

Homologous arm name	Homologous arm sequence
BCR/ABL1 Translocation	TGACGGGGCTTCGCCCATCTGGCATCTTTCTTTCTGCATCAAGGACTGCAAACCCGTTGGGCAGTGCCAGGGGATACCGAGGTGGATGTCCAGGCTTGGAGTGCCCAACTGATCAGCAGCATCTGCCCCAGGAATGGCAGGGGCAGGGGCAAAGCACAGGCATCTATCATCCTGCCTTAGCCACCATCAAGCCACCTCCCAGTTCCAACGAGCGGCTTCACTCAGACCCTGAGGCTCAAAGTCAGATGCTACTGGCCGCTGAAGGGCTTCTGGAAGAGAAAGGGGGAACAGAAAAAAGAAAAAGGAAGAGAAAATTGGGAGAAAAAATTAGTTTTATTCTCAGAAGCAAAAATATTTGTGTTTCCAACATTACACATTCCTATTTCATTTCTTAACATC
ABL1/BCR Translocation	GCTGCTCCTTTAACGAGCAAAGGGTGGTAGGTCAAACCGCAATTCCCAGATTTCTATCTCCTGCAACCACCACTAGCTGCCCACAACCCTTTTACCTTTAGTTATGCTTAGAGTGTTATCTCCACTGGCCACAAAATCATACAGTGCAACGAAAAGGTTGGGGTCATTTTCACTGGGTCCAGCGAGAAGGTTTTCCTTGGTTAACGTGCCTCGGATAGAAAGCTCTGAGTGAAGGGGTGCTGGTCAGACTCACGGTGACTTTTCTAGAGTCATTAGTCCTCCACCTTGCTTTGCAGCTAAGGCACCCAGGTGACCCCTCCTCAATGAGCAAGTGCCCCTCCACCAGGGCGAGGTCCTGGGGAGGATGCAGATCCAGGCCCCTGCCTTTGTGAAGCAACCA
VEGFA/PRNP Translocation (I-SceI insertion)	ACAGTGCATACGTGGGCTCCAACAGGTCCTCTTCCCTCCCAGTCACTGACTAACCCCGGAACCACACAGCTTCCCGTTCTCAGCTCCACAAACTTGGTGCCAAATTCTTCTCCCCTGGGAAGCATCCCTGGACACTTCCCAAAGGACCCCAGTCACTCCAGCCTGTTGGCTGCCGCTCACTTTGATGTCTGCAGGCCAGAATTACCCTGTTATCCCTATGGCGGCTACATGCTGGGAAGTGCCATGAGCAGGCCCATCATACATTTCGGCAGTGACTATGAGGACCGTTACTATCGTGAAAACATGCACCGTTACCCCAACCAAGTGTACTACAGGCCCATGGATGAGTACAGCAACCAGAACAACTTTGTGCACGACTGCGTCAATATCACAATCAAGCAGCACACGGTCACCACAA

RNF2/TRAC Translocation (I-SceI insertion)	CTTCATTGAGATTAGATTTCAACGTAGGAATTTTGG TGGGACACATACATTCAGACCATAGCACTTCCCTT CCAAATACTAAAATTGTTTTTCTCTCTTCTTTATTTC CAGCAATGTCTCAGGCTGTGCAGACAAACGGAAC TCAACCATTAAGCAAAACATGGAACTCAGTTTATA TGAGTTACAACGAACACCTCAGATTACCCTGTTATC CCTAACCAGCTGAGAGACTCTAAATCCAGTGACAA GTCTGTCTGCCTATTCACCGATTTTGATTCTCAAAC AAATGTGTCACAAAGTAAGGATTCTGATGTGTATAT CACAGACAAAACTGTGCTAGACATGAGGTCTATGG ACTTCAAGAGCAACAGTGCTGTGGCCTGGAGCAA CAAATCTGACTTTGCATGTGCAAACGCC
HEK3/HEK4 Translocation (I-SceI insertion)	GCAATTAGTCTATTTCTGCTGCAAGTAAGCATGCAT TTGTAGGCTTGATGCTTTTTTTCTGCTTCTCCAGCC CTGGCCTGGGTCAATCCTTGGGGCCCAGACTGAG CACGTGATGGCAGAGGAAAGGAAGCCCTGCTTCC TCCAGAGGGCGTCGCAGGACAGCTTTTCCTAGAC AGGGGCTAGTATGTGCAGCTCCTGCAATTACCCTG TTATCCCTATGGGGGTTAAAGCGGAGACTCTGGTG CTGTGTGACTACAGTGGGGGCCCTGCCCTCTCTG AGCCCCCGCCTCCAGGCCTGTGTGTGTGTCTCCG TTCGGGTTGAAAGGAGCCCGGGAAAAAGGCCCCA GAAGGAGTCTGGTTTTGGACGTCTGACCCCACCC CTCCCGCTTAGGGCTTCTGATCCCCCAGGGTGATT TCA
VEGFA/PRNP Translocation (VEGFA primer insertion)	ACAGTGCATACGTGGGCTCCAACAGGTCCTCTTCC CTCCCAGTCACTGACTAACCCCGGAACCACACAG CTTCCCGTTCTCAGCTCCACAAACTTGGTGCCAAA TTCTTCTCCCCTGGGAAGCATCCCTGGACACTTCC CAAAGGACCCCAGTCACTCCAGCCTGTTGGCTGC CGCTCACTTTGATGTCTGCAGGCCAGACTCCAGAT GGCACATTGTCAGAGTGGCGGCTACATGCTGGGA AGTGCCATGAGCAGGCCCATCATACATTTCGGCAG TGACTATGAGGACCGTTACTATCGTGAAAACATGC ACCGTTACCCCAACCAAGTGTACTACAGGCCCATG GATGAGTACAGCAACCAGAACAACTTTGTGCACGA CTGCGTCAATATCACAATCAAGCAGCACACGGTCA CCACAA
RNF2/TRAC Translocation (TRAC primer insertion)	CTTCATTGAGATTAGATTTCAACGTAGGAATTTTGG TGGGACACATACATTCAGACCATAGCACTTCCCTT CCAAATACTAAAATTGTTTTTCTCTCTTCTTTATTTC CAGCAATGTCTCAGGCTGTGCAGACAAACGGAAC TCAACCATTAAGCAAAACATGGAACTCAGTTTATA

	TGAGTTACAACGAACACCTCAGCCCACAGATATCC AGAACCCTGACCAGCTGAGAGACTCTAAATCCAGT GACAAGTCTGTCTGCCTATTCACCGATTTTGATTCT CAAACAAATGTGTCACAAAGTAAGGATTCTGATGT GTATATCACAGACAAAACTGTGCTAGACATGAGGT CTATGGACTTCAAGAGCAACAGTGCTGTGGCCTG GAGCAACAAATCTGACTTTGCATGTGCAAACGCC
Chr11 HBB/FANCF 16.8MB Knock out	GGTCCAAGGGTAGACCACCAGCAGCCTAAGGGTG GGAAAATAGACCAATAGGCAGAGAGAGTCAGTGC CTATCAGAAACCCAAGAGTCTTCTCTGTCTCCACA TGCCCAGTTTCTATTGGTCTCCTTAAACCTGTCTTG TAACCTTGATACCAACCTGCCCAGGGCCTCACCAC CAACTTCATCCACGTTCACCTTGCCCCGCTGCAGA AGGGATTCCATGAGGTGCGCGAAGGCCCTACTTC CGCTTTCACCTTGGAGACGGCGACTCTCTGCGTA CTGATTGGAACATCCGCGAAATGATACGCCTCTCT GCAATGCTATTGGTCGAAATGCATGTCAATCTCCCA GCGTCTTTATCCGTGTTCCTTGACTCTGGGCAACT TAAAAGCCCTAATACTTT
VEGFA/PRNP Translocation (I-SceI insertion) HA-misaligned 50bp	AGGGCCTCTGTCTGCCCAGCTGCCTCCCCCTTTG GGTTTTGCCAGACTCCACAGTGCATACGTGGGCT CCAACAGGTCCTCTTCCCTCCCAGTCACTGACTAA CCCCGGAACCACACAGCTTCCCGTTCTCAGCTCC ACAAACTTGGTGCCAAATTCTTCTCCCCTGGGAAG CATCCCTGGACACTTCCCAAAGGACCCCATTACCC TGTTATCCCTAGCAGTGACTATGAGGACCGTTACTA TCGTGAAAACATGCACCGTTACCCCAACCAAGTGT ACTACAGGCCCATGGATGAGTACAGCAACCAGAAC AACTTTGTGCACGACTGCGTCAATATCACAATCAA GCAGCACACGGTCACCACAACCACCAAGGGGGA GAACTTCACCGAGACCGACGTTAAGATGATGGAG CGCGT
VEGFA/PRNP Translocation (I-SceI insertion) HA-misaligned 100bp	CTGGCCTCAGTTCCCTGGCAACATCTGGGGTTGG GGGGGCAGCAGGAACAAGGGCCTCTGTCTGCCC AGCTGCCTCCCCCTTTGGGTTTTGCCAGACTCCA CAGTGCATACGTGGGCTCCAACAGGTCCTCTTCC CTCCCAGTCACTGACTAACCCCGGAACCACACAG CTTCCCGTTCTCAGCTCCACAAACTTGGTGCATTA CCCTGTTATCCCTAAACCAAGTGTACTACAGGCCC ATGGATGAGTACAGCAACCAGAACAACTTTGTGCA CGACTGCGTCAATATCACAATCAAGCAGCACACGG TCACCACAACCACCAAGGGGGAGAACTTCACCGA

	GACCGACGTTAAGATGATGGAGCGCGTGGTTGAG CAGATGTGTATCACCCAGTACGAGAGGGAATCTCA GGCCTAGTACC
VEGFA/PRNP Translocation (I-SceI insertion) HA-misaligned 200bp	GTGGGAGCTCTGGGCAGCTGGCCTACAGACGTTC CTTAGTGCTGGCGGGTAGGTTTGAATCATCACGCA GGCCCTGGCCTCCACCCGCCCCCACCAGCCCCC TGGCCTCAGTTCCCTGGCAACATCTGGGGTTGGG GGGGCAGCAGGAACAAGGGCCTCTGTCTGCCCA GCTGCCTCCCCCTTTGGGTTTTGCCAGACTCCATT ACCCTGTTATCCCTACCACCAAGGGGGAGAACTTC ACCGAGACCGACGTTAAGATGATGGAGCGCGTGG TTGAGCAGATGTGTATCACCCAGTACGAGAGGGAA TCTCAGGCCTATTACCAGAGAGGATCGAGCATGGT CCTCTTCTCCTCTCCACCTGTGATCCTCCTGATCT CTTTCCTCATCTTCCTGATAGTGGGATGAGGAAGG TCTTCC
