# Supplementary figures and images for: Recruitment and rejoining of remote double-strand DNA breaks for enhanced and precise chromosome editing
Source: Genome Biol. 2025 Mar 11;26:53. doi: 10.1186/s13059-025-03523-8 (PMC11895233; doi:10.1186/s13059-025-03523-8)

Fig1

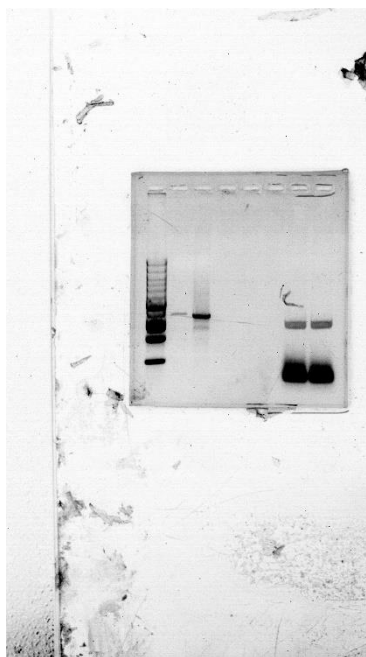

Fig2

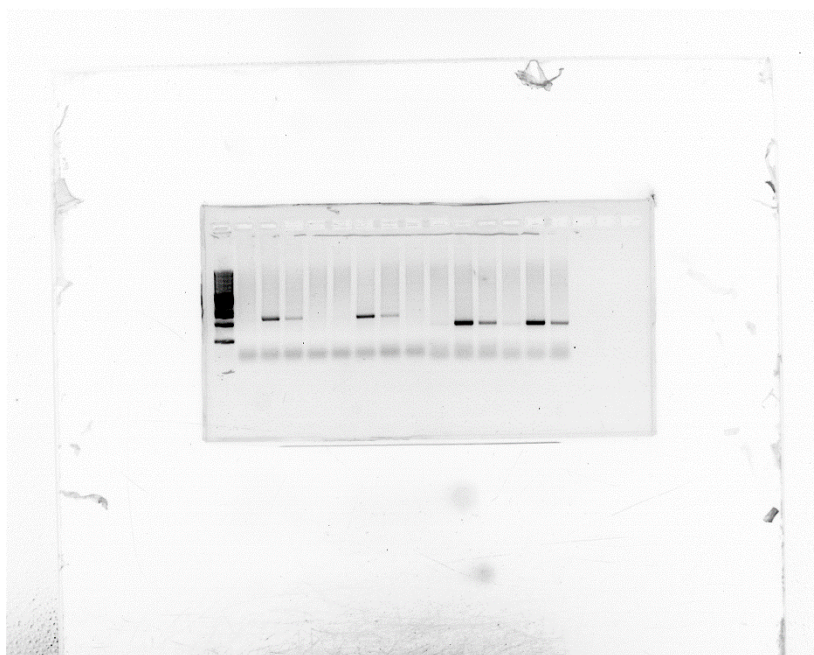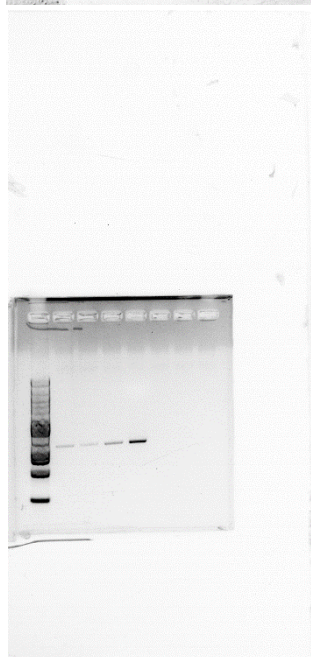

Fig4

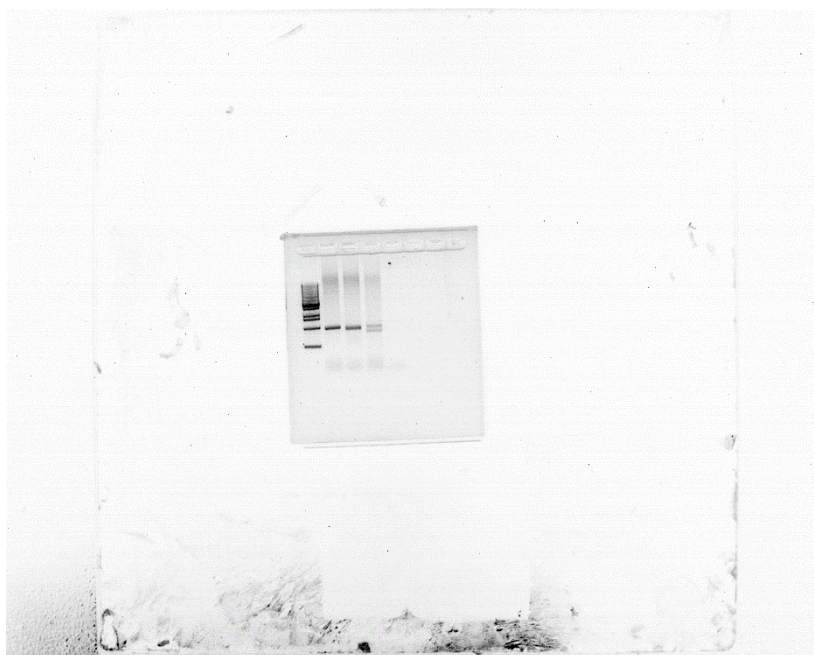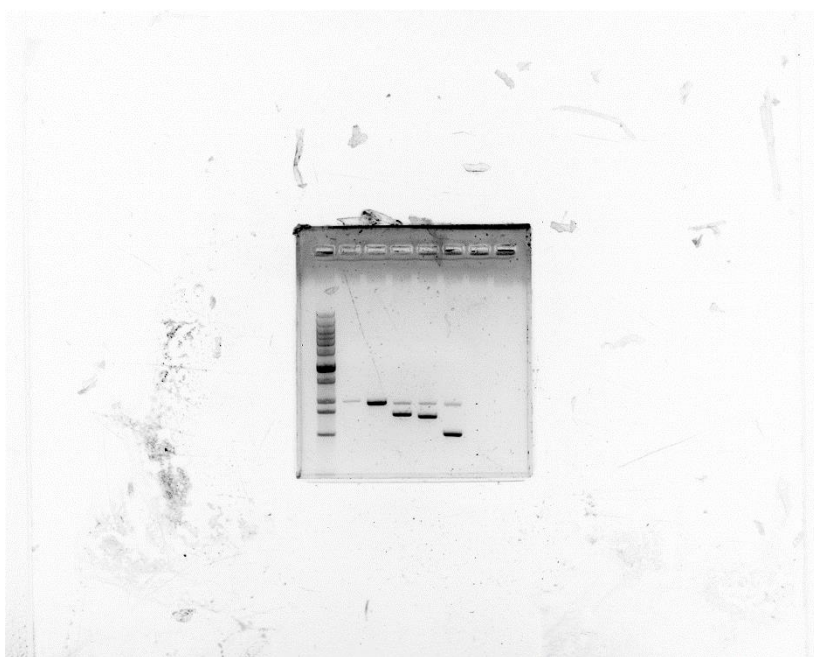

FigS1

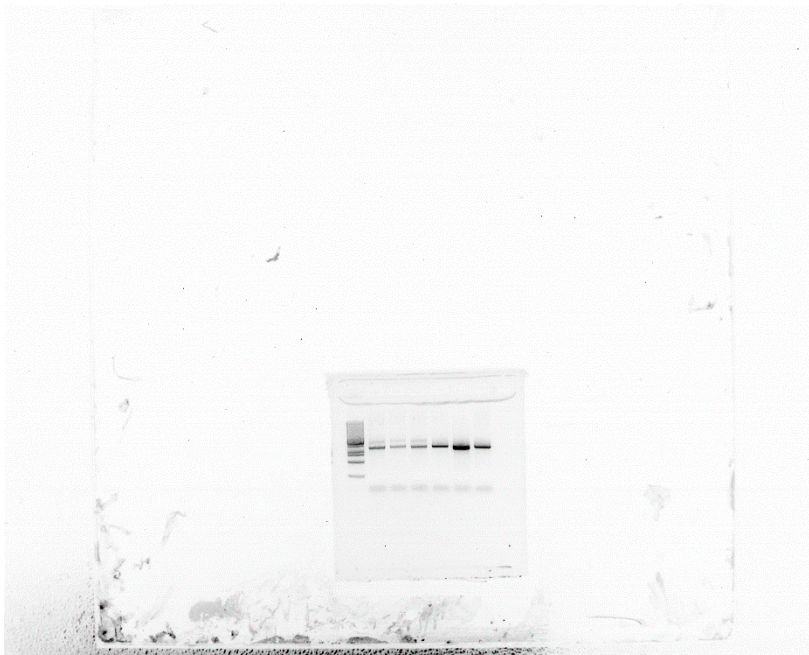

FigS4-1

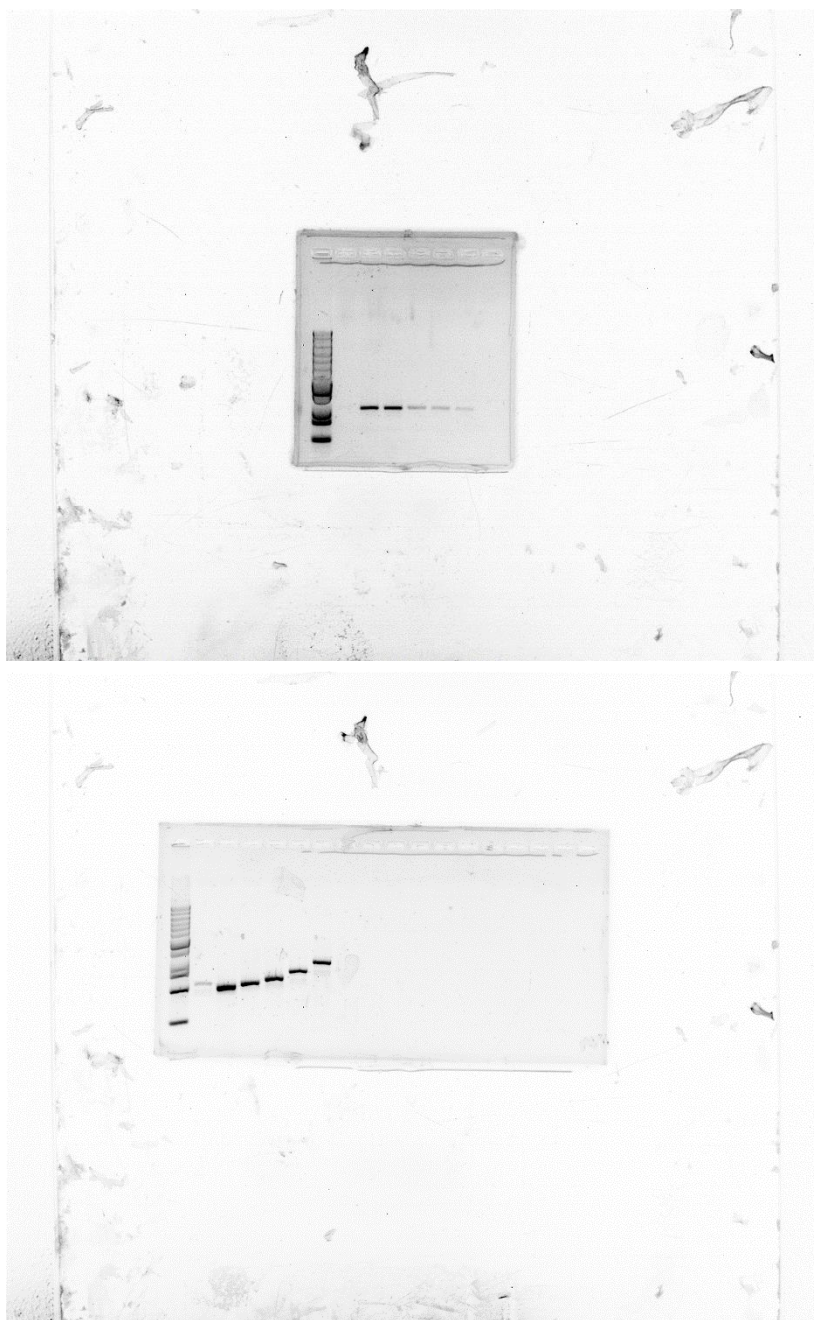

FigS6

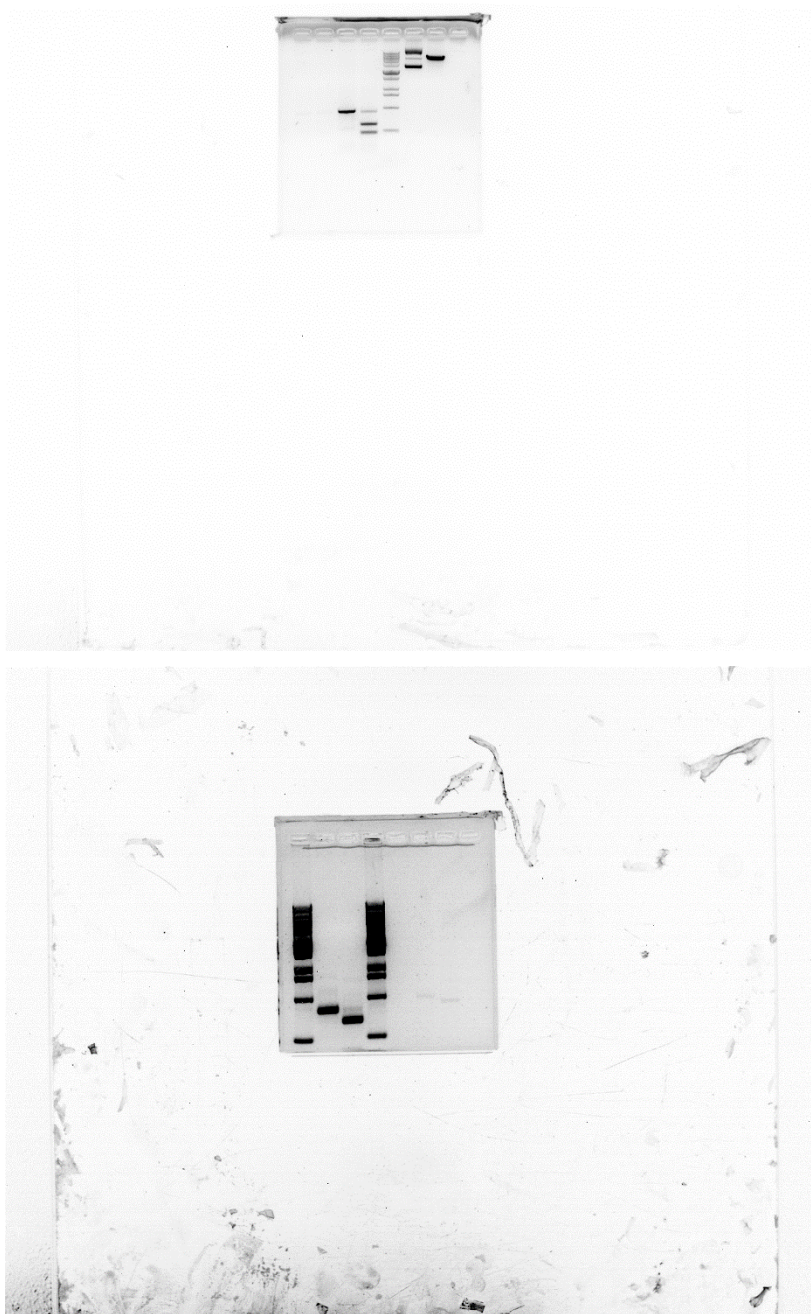

Supplement: Supplementary file 3 — Additional file 3. Uncropped DNA gel electrophoresis images. [file 13059_2025_3523_MOESM3_ESM.pdf]
